# *TERT* promoter mutations and telomere length in adult malignant gliomas and recurrences

**DOI:** 10.18632/oncotarget.3329

**Published:** 2015-03-12

**Authors:** Barbara Heidenreich, P. Sivaramakrishna Rachakonda, Ismail Hosen, Florian Volz, Kari Hemminki, Astrid Weyerbrock, Rajiv Kumar

**Affiliations:** ^1^ Division of Molecular Genetic Epidemiology, German Cancer Research Center, Heidelberg 69120, Germany; ^2^ Department of Neurosurgery, University Medical Center Freiburg, Freiburg 79106, Germany; ^3^ Center for Primary Health Care Research, Lund University, Malmö, Lund 22100, Sweden

**Keywords:** Gliomas, *TERT* promoter, *IDH*, telomere length, *TERT* expression

## Abstract

In this report on 303 gliomas we show the highest frequency of *TERT* promoter mutations in gliobastomas (80%) followed by oligodendrogliomas (70%) and astrocytomas (39%). We observed positive association between *TERT* promoter and *IDH* mutations in oligodendroglial tumors (OR = 26.3; 95% CI 2.5–250.2) and inverse association in primary glioblastomas (OR = 0.13; 95% CI 0.03–0.58). Tumors with *TERT* promoter mutations compared to those without showed increased TERT transcription; we also showed difference in the transcription levels due to the two main mutations. Tumors with *TERT* promoter mutations had shorter telomeres than those without. The patients with only *TERT* promoter mutations showed worst survival (median survival 14.6 months) and patients with both *IDH* and *TERT* promoter mutations showed best survival (246.5 months). In patients with astrocytoma, the *TERT* promoter mutations only associated with poor survival (*P* < 0.0001); *IDH* mutations and 1p/19q deletions associated with increased survival (*P* = 0.0004). *TERT* promoter mutations in low grade gliomas associated with reduced progression free survival (HR 10.2; 95% CI 1.9 – 55.9). While our data affirm the role of *TERT* promoter mutations in glial tumors, effects on transcription and telomere length emphasise the importance of telomere biology in disease genesis and outcome.

## INTRODUCTION

Gliomas are the most common primary malignant brain tumors in adults that mainly arise in glial tissue of the brain. Those tumors are either astrocytic, oligodendrocytic or a mixture of the two cell types and are typically categorized according to the International Classification of Diseases – Oncology, version 3 (ICD-O-3) and World Health Organization (WHO) grade [1, 2]. The most common gliomas are glioblastomas, comprising the two sub-types primary and secondary glioblastoma. The sub-types follow different modes of progression and show distinct genetic alterations [1, 3–5]. The majority is constituted by primary glioblastomas and those tumors develop quickly. Most of the patients present symptoms less than six months prior to diagnosis [3]. As a consequence, glioblastomas exhibit a poor prognosis with a 5-year relative survival of ~5% [1].

Challenging histopathological features of glial tumors and new possibilities in treatment add to the need of genetic markers with prognostic as well as predictive potential [6–10]. Mutations in Isocitrate dehydrogenase 1 (*IDH*1) and - less common - Isocitrate dehydrogenase 2 (*IDH*2) are examples for genetic alterations with prognostic value and potential as novel therapeutic targets [9, 11]. As such, *IDH* (*IDH*1 and *IDH*2) mutations are associated with prolonged progression-free survival and seem to indicate patients that might benefit from chemoradiotherapy [12]. Those mutations are common within gliomas that follow astrocytic progression (astrocytomas, oligoastrocytomas and secondary glioblastomas) but are rare in primary glioblastomas [13, 14]. Co-deletions of chromosome 1p and 19q have also been demonstrated to associate with better survival rates and furthermore correlate with *IDH* mutations, representing a typical feature of the oligodendroglial subtype [14, 15].

Reports on mutations within the core promoter of the telomerase reverse transcriptase (*TERT*) gene in different cancers have consistently shown occurrence at highest frequencies in adult gliomas [16–21]. *TERT* encodes the rate-limiting catalytic subunit of telomerase, which is involved in de novo addition of telomere repeats at chromosomal ends. The discovery of the *TERT* promoter mutations provides a possibility for (i) gaining insight into mechanistic questions in glioma development and (ii) a new biomarker and eventual therapeutic target [22]. The *TERT* promoter mutations exert influence through increased expression due to creation of new binding motifs for Ets/TCF transcription factors [23]. Recent reports indicate a clear subtype-specific distribution of the *TERT* promoter mutation in different gliomas, which in combination with *IDH* mutations allows classification according to their histological subgroups [24, 25].

In the present study, we investigated and show the occurrence and correlations between alterations at *CDKN2A/B*, 1p/19q, *IDH* and the *TERT* promoter mutations in different histological sub-types of gliomas and their effect on patient survival. We also show that the presence of *TERT* promoter mutations influences telomere length and affect the gene expression.

## RESULTS

### Patient characteristics

A total of 303 gliomas and 22 recurrences from patients treated at the Department of Neurosurgery of the University Medical Center Freiburg were investigated. The 303 primary tumors comprised of 56 astrocytomas, 55 oligoastrocytomas, 27 oligodendrogliomas and 165 primary or *de novo* glioblastomas (Table 1). The patient group included 111 women and 192 men with a mean age of 53 ± 17 years (median 55; range: 8 – 85 years; Table 2; Supplementary Table 1).

**Table 1:** Frequencies of alterations at the *TERT* promoter, *IDH1*/*2*, 9p21 and 1p/19q in different glioma subtypes

	All	Astrocytomas	Oligoastrocytomas	Oligodendrogliomas	Primary Glioblastomas
	*n* = 303	*n* = 56	*n* = 55	*n* = 27	*n* = 165
***TERT* promoter mutations**	199/303 (65.7%)	22/56 (39.3%)	26/55 (47.3%)	19/27 (70.4%)	132/165 (80.0%)
***IDH1/2*** mutations	100/303 (33.0%)	37/56 (66.1%)	39/55 (71.0%)	16/27 (59.3%)	8/165 (4.9%)
**9p21** deletions	119/257 (46.3%)	17/47 (36.2%)	13/47 (27.7%)	9/25 (36.0%)	80/138 (58.0%)
**deletions at 1p/19q**	74/259 (28.6%)	15/49 (30.6%)	24/47 (51.1%)	14/25 (56.0%)	21/138 (15.2%)
deletions at 1p	12/259 (4.6%)	3/49 (6.1%)	0/47 (0%)	0/25 (0%)	9/138 (6.5%)
deletions at 19q	15/259 (5.8%)	2/49 (4.1%)	5/47 (10.6%)	0/25 (0%)	8/138 (5.8%)
1p/19q co-deletion	47/259 (18.1%)	10/49 (20.4%)	19/47 (40.4%)	14/25 (56%)	4/138 (2.9%)

**Table 2:** Distribution of *TERT* promoter mutations and association with genetic alterations in gliomas

	All *n* = 303	TERT promoter		
		wt	mut	
Gender				
male	192	72	120	OR = 1.48; 95% CI 0.8 – 2.45; *P* = 0.1
female	111	32	79	
Age				
≤ 55	152	78	74	OR = 5.07; 95% CI 2.99 – 8.60; ***P* < 0.0001**
> 55	151	26	125	
Grade				
low (II)	78	42	36	OR = 3.05; 95% CI 1.79 – 5.19; ***P* < 0.0001**
high (III + IV)	225	62	163	
*IDH*				
wildtype	203	53	150	OR = 0.34; 95% CI 0.21 – 0.56; ***P* < 0.0001**
mutation	100	51	49	
9p21				
wildtype	138	60	78	OR = 3.04; 95% CI 1.74 – 5.33; ***P* < 0.0001**
mutation	119	24	95	
*missing*	*46*			
Deletions at 1p and/or 19q				
wildtype	185	73	112	OR = 3.37; 95% CI 1.70 – 6.68; ***P* = 0.0003**
deletion	74	12	62	
*missing*	*44*			
1p/19q codeletions				
wildtype	214	85	129	OR = 1.35; 95% CI 1.24 – 1.47; ***P* < 0.0001**
codeletion	45	0	45	
*missing*	*44*			

*P*-values were derived from χ^2^-test and considered statistically significant if < 0.05.

**Bold font** indicates statistical significance.

### *TERT* promoter mutations

Overall *TERT* promoter mutations were present in 199 of 303 (66%) gliomas. The –124C > T mutation was present in 147 tumors (74%), whereas –146C > T was found in 51 cases (26%) and one tumor carried the –124_125CC > TT tandem mutation. Primary glioblastomas had the highest frequency of *TERT* promoter mutations (132/165; 80%) followed by oligodendrogliomas (19/27; 70%); astrocytomas showed the lowest frequency, with 22 out of 56 (39%) samples showing mutations (Table 1). A difference in the frequency of mutations in tumors from women and men was not statistically significant. *TERT* promoter mutations were more frequent in patients older than 55 years of age at diagnosis (OR = 5.07; 95% CI 2.99 – 8.60; *P* < 0.0001). Also, *TERT* promoter mutations were found associated with high grade (grade III + IV) tumors when compared to lower grade (grade II) lesions (OR = 3.05; 95% CI 1.79 – 5.19; *P* < 0.0001; Table 2).

### *TERT* promoter mutations and alterations in other genes

Besides *TERT* promoter mutations, we investigated alterations at *CDKN2A/B*, *IDH1*, *IDH2* and chromosome arms 1p and 19q. *IDH1* or *IDH2* mutations (‘*IDH* mutations’) were present in 100 of 303 (33%) glioma tumors (Table 1). The c.395G > A (R132H) mutation in *IDH*1 was the most common one, present in 94 cases (94%); two gliomas harbored the *IDH1* c.394C > T mutation (R132H). *IDH2* mutations were found in four tumors (4%), with c.515G > A (R172K) in three tumors and c.515G > T (R172M) in one tumor. The highest frequency of *IDH* mutations was detected in oligoastrocytomas (39/55; 71%), followed by astrocytomas (37/56; 66%) and oligodendrogliomas (16/27; 59%) (Table 1).

**Figure 1:**
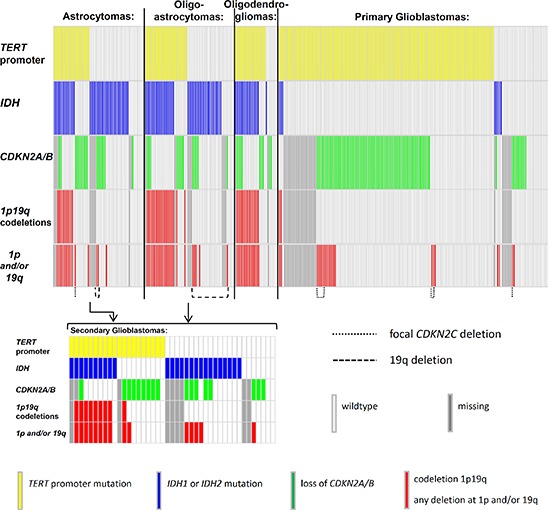
Distribution of mutations in gliomas The distribution of mutations in the *TERT* promoter, *IDH*1 and *IDH*2 (*IDH*) and deletions at 9p21 (*CDKN2A/B*), 1p and 19q. Mutations are indicated in different colours. *TERT* = telomerase reverse transcriptase; *IDH*1 = isocitrate dehydrogenase 1; *IDH*2 = isocitrate dehydrogenase 2; *CDKN2A* = cyclin-dependent kinase inhibitor 2A; *CDKN2B* = cyclin-dependent kinase inhibitor 2B; *CDKN2C* = cyclin-dependent kinase inhibitor 2C; chromosome arm 1p; chromosome arm 19q; wildtype = no mutation found within the investigated loci.

Data on deletions at 1p and/or 19q were available for 259 gliomas. 74 tumors had deletions at chromosome arms 1p and/or 19q, of which 47 were co-deletions at both loci. The highest frequency of 1p/19q co-deletions was in oligodendrogliomas with 14/28 (56%), followed by oligoastrocytomas 19/47 (40%). The *CDKN2C* (p18^INK4C^) locus at chromosome 1p showed focal deletions in nine gliomas, of which eight were primary glioblastomas and one was a grade II astrocytoma. Of these nine, six tumors were also deleted at the *CDKN2A/B* and eight tumors carried *TERT* promoter mutations (Figure 1).

In the entire set of tumors mutations in *IDH* and deletions at 1p and/or 19q were associated with an odds ratio (OR) of 9.97 (95% CI 5.39 – 18.46; *P* < 0.0001). *TERT* promoter mutations and deletions at 1p/19q when present tended to occur together (OR = 3.37; 95% CI 1.70 – 6.68; *P* = 0.0003) (Table 2). In contrast, *TERT* promoter mutations and *IDH* mutations were inversely correlated (OR = 0.34; 95% CI 0.21 – 0.56; *P* < 0.0001). However, in oligodendroglial tumors, *TERT* promoter and *IDH* mutations occurred together (OR = 26.25; 95% CI 2.46 – 250.20; *P* = 0.001). In primary glioblastomas the frequency of *IDH* mutations was low (8/165, 5%) and showed an inverse correlation (OR 0.13; 95% CI 0.03 – 0.58; *P* = 0.002) with *TERT* promoter mutations (132/165; 80%). In astrocytomas, where both, *TERT* promoter and *IDH* mutations, were frequent, the OR of 0.60 suggested an inverse association; however this observation was not statistically significant (Supplementary Table 2).

The *CDKN2A/B* locus was deleted in 119 of 257 (46%) gliomas. The highest frequency of deletions was observed in primary glioblastomas (80/138; 58%) (Table 1). Association between loss of *CDKN2A/B* and the *TERT* promoter mutations was statistically significant associated (OR = 3.04; 95% CI 1.74 – 5.33, *P* < 0.0001) (Supplementary Table 2).

### TERT mRNA expression levels

**Figure 2:**
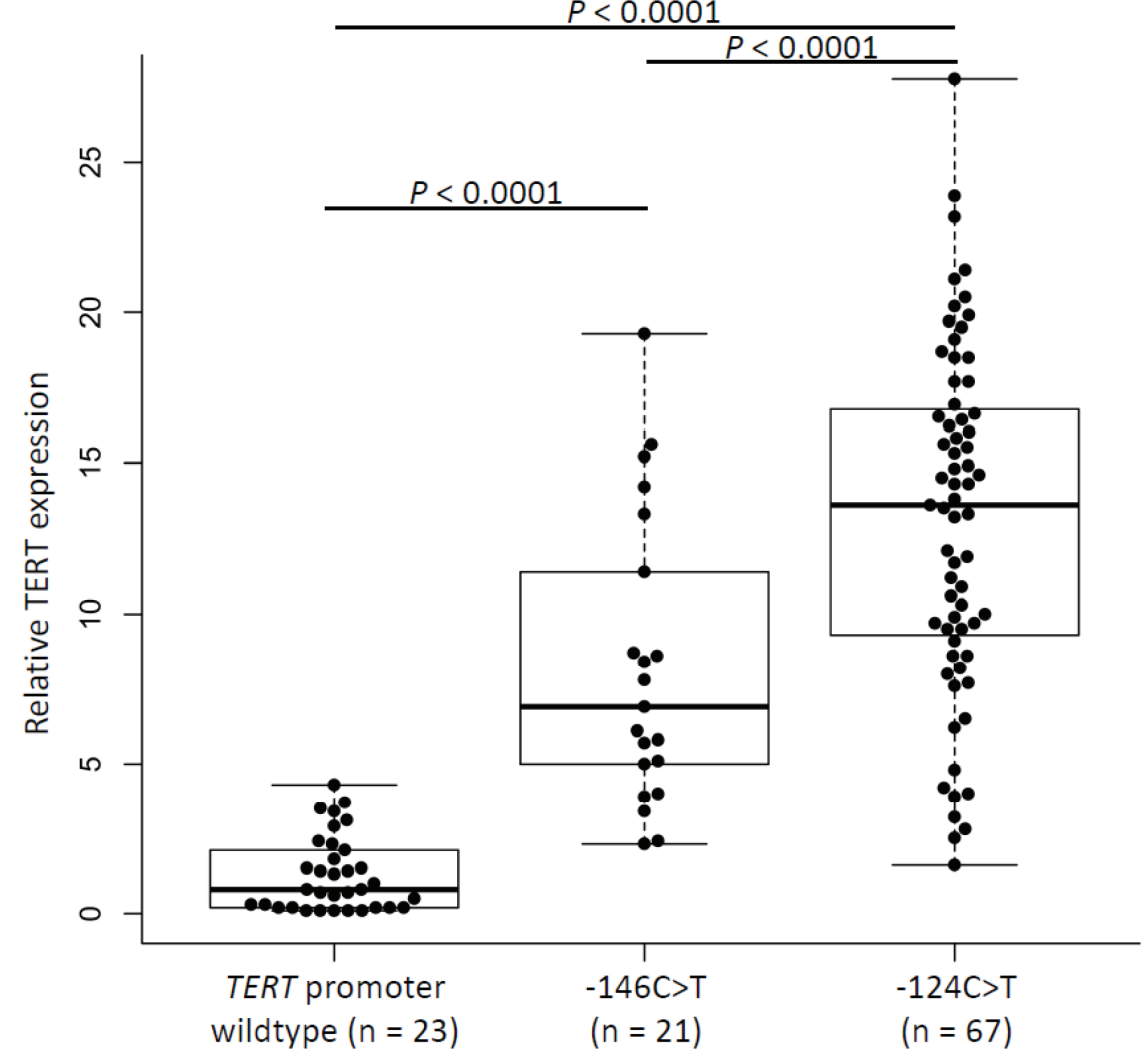
Relative TERT expression in glioma samples according to mutation status of the *TERT* promoter Comparison of TERT mRNA expression in gliomas without and with *TERT* promoter mutations –124C > T or –146C > T. Expression of *TERT* gene was normalized to GUSB expression, used as an internal standard, and quantification was performed by ΔΔC_T_ method with log2 transformation. Experiments were carried out in triplicates and box plots represent mean ± standard error of means; *P* (wildtype/–146C > T) < 0.0001; *P* (Wildtype/–124C > T) < 0.0001; *P* (–146C > T/–124C > T) < 0.0001. *P*-values were determined by *t*-test.

RNA was available from 111 gliomas, of which 88 carried *TERT* promoter mutations (–124C > T *n* = 67; –146C > T *n* = 21) and 23 were without the mutations. Analysis of quantitative real-time PCR data showed statistically significant higher levels of mRNA in gliomas with *TERT* promoter mutations than in wildtype tumors (*P* < 0.0001, *t*-test; Figure 2). Tumors harboring the –124C > T mutation had a 14-fold increase in mRNA expression compared to wildtype lesions, whereas –146C > T tumors displayed a 7-fold increase. The difference in expression between tumors with –124C > T and –146C > T mutation was statistically significant (*P* < 0.0001, *t*-test; Figure 2).

### Relative telomere length and correlation with *TERT* promoter mutations

**Figure 3:**
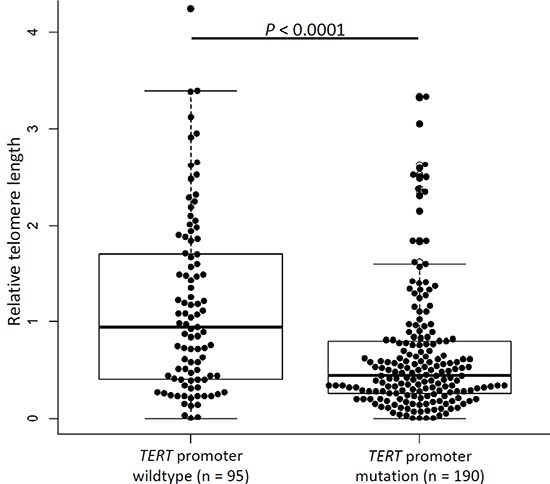
Telomere lengths in glioma tumors Relative telomere length in gliomas without and with *TERT* promoter mutations. Experiments were carried out in triplicate and box plots represent mean ± s.e.m. *P*-values were determined by *t*-test.

Results from measurement of relative telomere length were available for 285 gliomas of which 190 were with and 95 were without *TERT* promoter mutations. Relative telomere length ranged between 0.01 and 4.24 with a median value of 0.53. Tumors carrying *TERT* promoter mutations had shorter telomeres (median 0.44) compared to tumors without mutations (median 0.94, *P* < 0.0001, *t*-test; Figure 3). Stratification of the data according to age showed that the differences in relative telomere lengths in tumors with *TERT* promoter mutations was statistically significant in patients with age ≤ 52 years at diagnosis (*P* < 0.0001, *t*-test; Supplementary Figure 1) but not in patients with age > 52 (*P* = 0.3). Stratification of the data according to the combined *IDH* and *TERT* promoter status revealed that only in the two groups that carry *TERT* promoter mutations (*TERT* promoter mutated and either *IDH* mutated or wildtype) telomere lengths is statistically significant shorter than in tumors that carry neither of the mutations (Supplementary Figure 2).

### Correlation between relative telomere length and TERT mRNA expression

We measured correlation between relative telomere length and TERT mRNA levels in 88 tumors for which both data were available. Data analysis showed a statistically significant inverse correlation between relative telomere length and TERT expression at mRNA level (R = –0.24; *P* = 0.02; Supplementary Figure 3).

### Effect of *TERT* promoter mutations and alterations in other genes on survival

To assess the effect of the investigated alterations, we conducted a survival analysis excluding those patients with incomplete mutation data (*n* = 46); further, patients aged younger than 20 years at diagnosis (*n* = 7) were excluded from the analysis.

**Figure 4:**
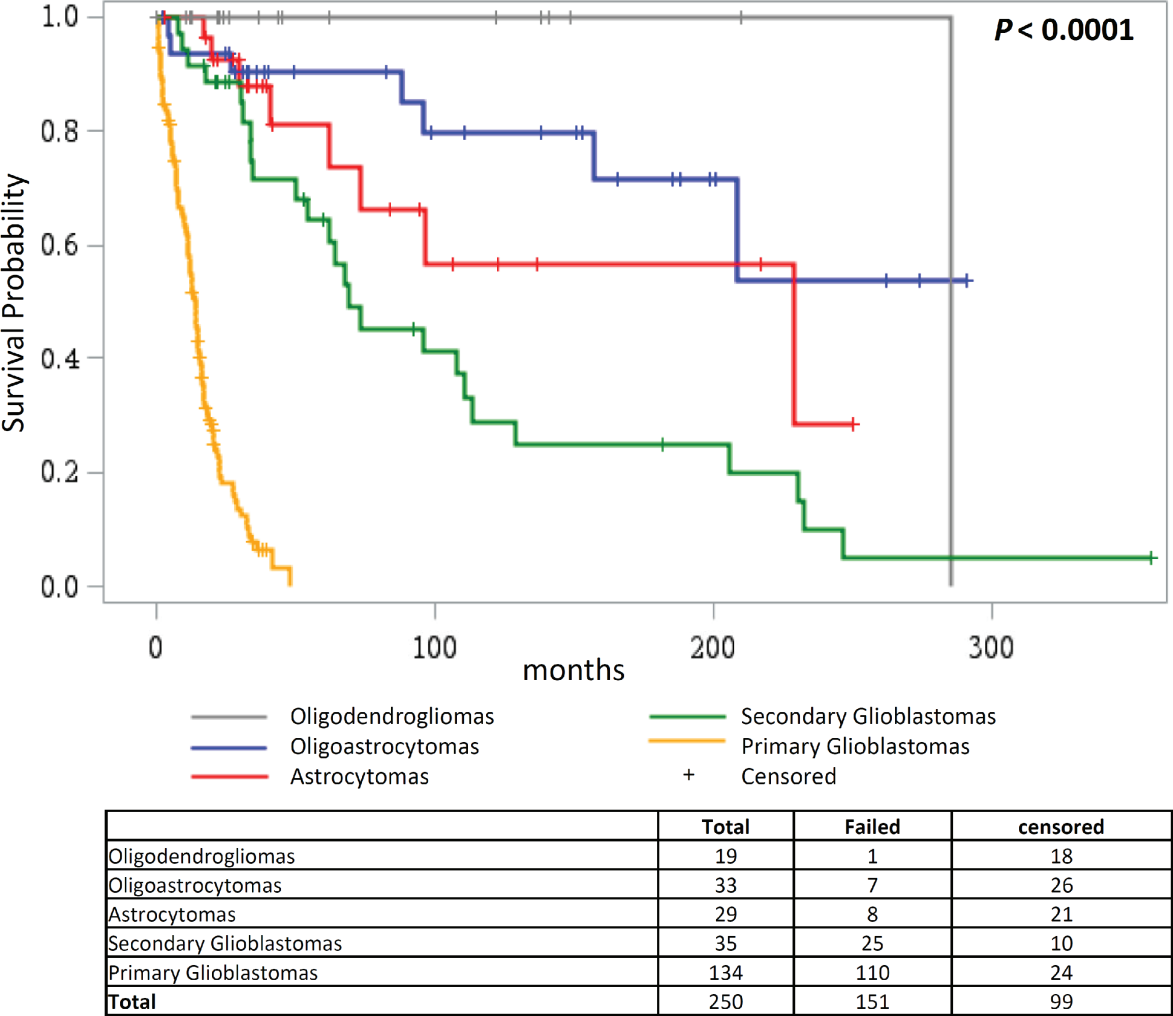
Overall survival according to glioma histology Kaplan Meier analysis of differences in overall survival in glioma patients according to their histology.

Survival analysis of the different histological subgroups of glioma showed that patients suffering from primary glioblastomas had worst overall survival (median survival 14.0 months), followed by secondary glioblastomas (median survival 69.1 months); diagnoses of oligoastrocytomas, oligodendrogliomas and astrocytomas were associated with better survival than primary and secondary glioblastomas (*P* < 0.0001; Figure 4).

**Figure 5:**
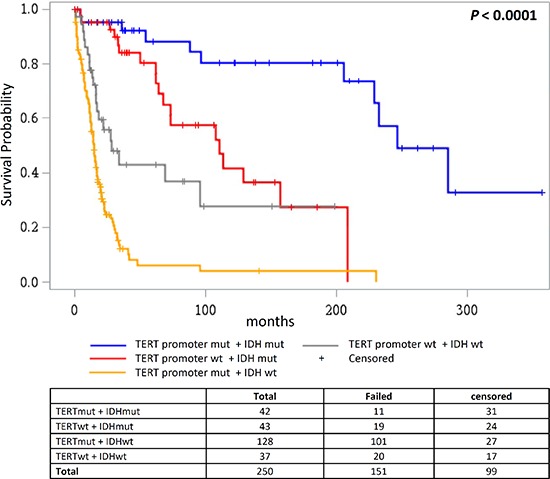
Overall survival according to combined status of *TERT* promoter and *IDH* mutations Overall survival in glioma patients where patient groups are defined by the mutational status of the *TERT* promoter and *IDH*1/*IDH*2.

Stratification of patients based on the mutational status of the *TERT* promoter and *IDH* resulted in four groups with differing overall survival (*P* < 0.0001; Figure 5). The group with *TERT* promoter mutations and without *IDH* mutations showed worst overall survival (median survival 14.6 months), followed by the two groups without *TERT* promoter mutations that were either wildtype for *IDH* (median survival 28.5 months) or carried *IDH* mutations (median survival 110.6 months). Best overall survival was associated with the presence of both *TERT* promoter and *IDH* mutations (median survival 246.5 months), which resembles oligodendroglial progression. A multivariate model that included age, grade, treatment and loss of *CDKN2A/B* showed that the combined presence of *TERT* promoter and *IDH* mutations was the only independent factor (HR = 0.22; 95% HR CI 0.082 – 0.59; *P* = 0.03) (Supplementary Table 3). The adverse effect of only *TERT* promoter mutations was statistically significant in a model that did not include tumor grade and age (HR = 2.18; 95% HR CI 1.31 – 3.63; *P* = 0.003).

In survival analysis in patients with primary glioblastomas we did not observe any effect of the *TERT* promoter mutations. Both, patients with and without *TERT* promoter mutations, had a median survival of 14.0 months (Supplementary Figure 4). The multivariate model that included age, treatment, *IDH* mutations, 1p/19q codeletions and *CDKN2A* deletions also did not show an effect of the *TERT* promoter mutations on patient survival (Table 3).

**Table 3:** Multivariate Model in primary Glioblastomas

Parameter		*P*	HR	95% CI	
*TERT* promoter	Mutation	0.33	0.77	0.45	1.31
*IDH*	Mutation	0.16	0.43	0.13	1.41
1p/19q co-deletion	Deletion	0.61	1.15	0.66	2.00
*CDKN2A/B*	Deletion	0.77	0.94	0.62	1.43
Treatment - Group	RT	**0.03**	**2.09**	**1.07**	**4.08**
Treatment - Group	TMZ+ot	0.40	0.80	0.48	1.35
Treatment - Group	none	**< 0.0001**	**15.21**	**7.20**	**32.13**
Age		**0.01**	**1.03**	**1.01**	**1.05**

Patients with astrocytomas were also grouped according to their *TERT* promoter and *IDH* status as described before. We observed that the presence of only *TERT* promoter mutations was associated with worst survival (median survival 29.7 months), followed by the group with neither *TERT* promoter nor *IDH* mutations (median survival 96.2 months) and patients carrying only *IDH* mutations (median survival 107.9 months). *TERT* promoter mutations in combination with *IDH* mutations associated with best survival (median survival 229.3 months) (Supplementary Figure 5). A multivariate model that incorporated age, grade, treatment and loss of *CDKN2A/B* showed that in the group of astrocytomas, concomitant *TERT* promoter and *IDH* mutations indicate good survival (HR = 0.02; 95% HR CI 0.001 – 0.542; *P* = 0.02) whereas *TERT* promoter mutations alone associated with poor survival (HR = 37.7; 95% HR CI 1.63 – 870.8; *P* = 0.02) (Supplementary Table 4).

Further, we also investigated the independent effect of 1p/19q codeletions, *IDH* mutations and *TERT* promoter mutations in astrocytomas. Patients with 1p/19q codeletions were associated with better survival than patients without codeletions (96.2 months vs. 229.3 month, *P* = 0.0004, Supplementary Figure 6A). Similarly, patients with *IDH* mutations also showed better survival than without mutations (34.0 months vs. 205.8 months, *P* < 0.0001, Supplementary Figure 6B). Overall, the presence of *TERT* promoter mutations was not associated with an effect on survival (Supplementary Figure 6C); however, when patients with only *TERT* promoter mutations were considered the effect on poor survival was statistically significant (*P* < 0.0001; Supplementary Figure 6D). Here, patients with astrocytomas harboring *TERT* promoter mutations showed a median survival of 29.7 months compared to 107.9 months when mutations were not present.

A multivariate model that included *TERT* promoter mutations, *IDH* mutations, deletions at 1p/19q and *CDKN2A/B*, age, grade and treatment showed a protective effect for 1p/19q codeletions on patients' survival (HR = 0.002; 95% HR CI 0.0001 – 0.09; *P* = 0.002) as well as poor survival for patients with *TERT* promoter mutations (HR = 37.6; 95% HR CI 1.63 – 869.9; *P* = 0.02) and patients with *CDKN2A* deletions (HR = 6.1; 95% HR CI 1.5 – 25.4; *P* = 0.01) (Supplementary Table 5).

In the group of oligoastrocytomas we observed that the effect of the mutations followed a pattern similar to that observed in astrocytomas. 1p/19q codeletions were associated with better survival (*P* = 0.03, Supplementary Figure 7A). Patients with *IDH* mutations showed a median survival of 245.5 months compared to 230.3 months in patients wildtype for *IDH*; however, this difference was not statistically significant (Supplementary Figure 7B). *TERT* promoter mutations showed a statistically significant effect on poor survival only in patients without 1p/19q co-deletions (*P* = 0.005; Supplementary Figure 7C and 7D). The effect of the mutations or deletions in a multivariate model was not statistically significant (Supplementary Table 6 and 7).

We further stratified gliomas according to the disease grades, which resulted in one group with low grade gliomas that included 64 tumors with grade 2 astrocytomas, oligoastrocytomas and oligodendrogliomas. The second group comprised 185 high grade gliomas including primary glioblastomas and grade III/IV astrocytomas, oligoastrocytomas and oligodendrogliomas. Patients with low grade gliomas that showed 1p/19q codeletions had a median survival of 285.7 months compared to 209.1 months for patients wildtype for 1p/19q (*P* = 0.01; Supplementary Figure 8A). *IDH* mutations did not have a statistically significant effect on survival (Supplementary Figure 8B; Supplementary Table 8). The independent effect of *TERT* promoter mutations without the concurrent 1p/19q deletions could not be assessed (Supplementary Figure 8C; Supplementary Table 8).

A multivariate model on progression-free survival in low grade gliomas showed that *TERT* promoter mutations are associated with poor survival (HR = 10.2; 95% HR CI 1.9 – 55.9; *P* = 0.007) and 1p/19q codeletions have a protective effect (HR = 0.03; 95% HR CI 0.004 – 0.173; *P* = 0.0001) (Table 4).

**Table 4:** Multivariate Model for progression-free survival in Low Grade Gliomas

Parameter		*P*	HR	95% CI	
*TERT* promoter	Mutation	**0.01**	**10.21**	**1.87**	**55.87**
*IDH*	Mutation	0.10	2.03	0.87	4.73
1p/19q co-deletion	Deletion	**0.0001**	**0.03**	**0.004**	**0.17**
*CDKN2A/B*	Deletion	0.43	1.35	0.64	2.85
Treatment - Group	CCNU	0.43	0.60	0.17	2.14
Treatment - Group	Carmustin	0.23	3.89	0.42	36.28
Treatment - Group	PC	0.16	1.70	0.81	3.56
Treatment - Group	none	**0.005**	**0.22**	**0.08**	**0.63**
Age		0.81	1.00	0.97	1.04

In high grade gliomas *IDH* mutations were associated with better survival (median survival wt: 15.0 vs. median survival mut: 64.5 months, *P* < 0.0001; Supplementary Figure 9A); also 1p/19q co-deletions associated with better survival (*P* < 0.0001; Supplementary Figure 9B). *TERT* promoter mutations entailed poorer survival (median survival of 15.2 months) compared to patients without mutations (30.5 months, *P* = 0.001; Supplementary Figure 9C). A multivariate model that included *TERT* promoter and *IDH* mutations, deletions at 1p/19q and *CDKN2A/B*, treatment and age did not show statistically significant association of survival with any of the genetic alterations (Supplementary Table 9). However, exclusion of age from the model revealed association with *TERT* promoter mutations (HR = 2.25; 95% HR CI 1.29 – 3.94; *P* = 0.005), *IDH* mutations (HR = 0.42; 95% HR CI 0.19 – 0.91; *P* = 0.03) and 1p/19q codeletions (HR = 0.12; 95% HR CI 0.023 – 0.59; *P* = 0.01).

A multivariate model on progression-free survival in high grade gliomas showed no statistically significant effect of *TERT* promoter mutations but revealed a protective effect for *IDH* mutations as well as for 1p/19q deletions (Table 5).

**Table 5:** Multivariate Model on progression-free survival in High Grade Gliomas

Parameter		*P*	HR	95% CI	
*TERT* promoter	Mutation	0.39	1.27	0.73	2.21
*IDH*	Mutation	**0.01**	**0.36**	**0.16**	**0.82**
1p/19q co-deletion	Deletion	**0.02**	**0.09**	**0.01**	**0.71**
*CDKN2A/B*	Deletion	0.65	1.10	0.74	1.64
Treatment	BEV	0.55	1.33	0.53	3.33
Treatment	CCNU	0.15	2.47	0.71	8.54
Treatment	Carmustin	0.08	0.55	0.28	1.09
Treatment	PC	0.76	1.37	0.19	10.00
Treatment	none	0.13	0.57	0.27	1.18

**Figure 6:**
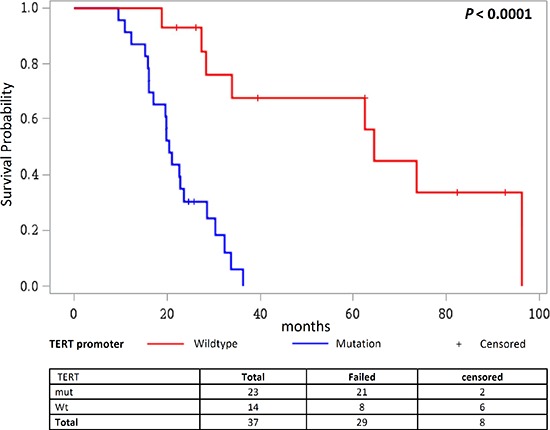
Overall survival in patients receiving TMZ and radiotherapy combined with a third adjuvant stratified according to the *TERT* promoter status Kaplan Meier analysis on overall survival in glioma patients receiving TMZ and radiotherapy combined with a third adjuvant, where patients are stratified according to their mutational status of the *TERT* promoter.

We further investigated the effect of *TERT* promoter mutations on overall survival according to different treatment regimens in patients with high grade gliomas. Therefore, patients were stratified into three groups, where group 1) did not receive any treatment, group 2) received radiotherapy alone or in combination with procarbazine or temozolomide and group 3) received temozolomide and radiotherapy combined with a third adjuvant. Patients who did not receive any treatment showed poor overall survival, followed by patients in group 2). In both groups the presence of *TERT* promoter mutations showed no effect. However, for patients in group 3) we observed that those without *TERT* promoter mutations (median survival 64.5 months) showed better overall survival than patients with the mutations (median survival 22.2 months; *P* = 0.0001; Figure 6; Supplementary Figure 10A). The same trend was also observed when patients with only primary glioblastomas were analyzed (*P* = 0.0001; Supplementary Figure 10B). Here, patients in group 3) showed better survival than patients in group 1) or 2) and again, especially patients with *TERT* promoter wildtype tumors (median survival 28.0 months) benefitted from the combination therapy (temozolomide + radiotherapy + adjuvant) in comparison to patients wildtype for the *TERT* promoter (median survival 20.1 months).

### Comparison between alterations in glioma tumors and matched recurrences

In total, 22 recurrences matched to 20 glioma tumors were available. In 18 cases, material from the patients' initial tumor and one recurrence was accessible and in another two cases tumor material from the initial lesion and two recurrences each was available (Supplementary Table 10). In addition to the described genetic alterations (*TERT* promoter, *IDH*1/2, *CDKN2A*/B deletions, 1p/19q codeletions) we included data on rs2853669, a common SNP within the investigated fragment of the *TERT* promoter, in order to serve as an internal control that tissues were acquired from the same patients. In 15 cases, glioma tumors and their matched recurrences were consistent in all of the investigated alterations. In contrast, in another 5 pairs, tumors varied in one or more alterations, when compared to their recurrences. In four pairs, an initial *CDKN2A/B* deletion could not be detected in the recurrences. For two pairs the initial *TERT* promoter mutations and one *IDH*1 mutation were not found in the recurrences and those also showed differences in their histology (Supplementary Table 10).

We also assessed survival in patients with initial tumors and recurrences based on *TERT* promoter mutation status. Two patients (BT-163 and BT-171) that had *TERT* promoter mutations in the initial tumors but not in the recurrence had median progression free survival of 65.3 months compared to 12.3 months for those patients (*n* = 3) that did not have the mutations in both initial tumors and recurrences. The median progression free survival for patients (*n* = 15) that had *TERT* promoter mutations in both initial tumors and recurrences was 8.5 months. The difference in progression free survival in three categories was, however, not statistically significant (*P* = 0.29; Supplementary Figure 11A). We also found a similar difference in overall survival in patients with recuurences based on mutational status of the *TERT* promoter. While the patients without mutations in initial tumors and recurrences showed overall median survival of 30.5 months compared to 16.2 months in patients with *TERT* promoter mutations in both. The patients with mutations in initial tumors and no mutations in the recurrence had better survival than the other two categories; however, overall difference was not statistically significant (Supplementary Figure 11B).

## DISCUSSION

Over the years different studies have demonstrated that the classification of gliomas based on their molecular profiles, in particular with inclusion of *IDH* mutations, results in subgroups with prognostic significance [13, 14, 26]. The discovery of mutations in the *TERT* promoter and subsequent reports of their occurrence at a high frequency in gliomas has resulted in further refinement of glioma classification [23, 24]. In this study, carried out on different sub-types of gliomas, we corroborated that the distribution of the *TERT* promoter and *IDH* mutations follow histological classification and predict the prognostic outcome of the disease, which, depending on the histological sub-type, is further influenced by deletions at 1p/19q loci [27]. Besides reporting a high frequency of *TERT* promoter mutations in different glioma types, we describe the correlations with *IDH* mutations and deletions at 1p, 19q and *CDKN2A/B* loci that have prognostic implications. Although the two main *TERT* promoter mutations create the exact same consensus sites for Ets/TCF transcription factors, our data suggest differences in the levels of increased *TERT* expression based on the mutational position [28]. Moreover, the presence of *TERT* promoter mutations strongly demonstrates the mechanistic relevance of telomere biology in cancer progression, which is predicated on the finding of shorter telomeres in tumors with mutations than without mutations.

The distribution of *TERT* promoter mutations in different glial tumors followed the reported pattern with highest frequencies being present in primary glioblastomas, followed by oligodendrogliomas, oligoastrocytomas and astrocytomas [17, 24, 25, 29–35]. The divergence between primary and secondary glioblastomas, considered as two distinct entities, was supported strongest by the incidence of *IDH* mutations, which are presumed as early initiating events in the development of secondary glioblastomas either via astrocytic or oligoastrocytic progression. The *IDH* mutations are conspicuously rare in primary glioblastomas [13, 14]. The high frequency of *TERT* promoter mutations in primary glioblastomas and an inverse association between *IDH* and *TERT* promoter mutations in astrocytomas and secondary glioblastomas suggests that those patterns of mutations represent astrocytic progression. The distinctive alteration in oligodendrogliomas is the combined loss of 1p and 19q [6, 15, 36]. Within this group, as reported earlier, we found that the *TERT* promoter mutations correlated with 1p/19q codeletions and *IDH* mutations [24, 25]. Those observations, in combination with previous reports, lead to the conclusion that co-occurrence of mutations in *IDH* and the *TERT* promoter reflect a mutational signature of oligodendrogliomas, whereas the presence of either *TERT* promoter or *IDH* mutations is a feature of astrocytic progression [25, 37].

In a set of available recurrences we found that in majority genotypes corresponded with the matched primary tumors. However, the two pairs with divergent status of *TERT* promoter showed mutations in the primary lesion but not in the recurrences. Generally, variations in genotypes can be ascribed either to detection limits of applied methods influenced by tumor heterogeneity, or varying genotypes in primary and recurrent tumor. An earlier report in primary glioma tumors and recurrences showed that recurrent tumors are often seeded by cells derived from the initial tumor at a very early stage of their evolution, which ultimately leads to deviating genotypes in primary tumors and recurrences [38].

The distribution of genetic alterations in primary tumors according to the course of glial progression was also evident in data from survival analysis. The combined mutational status profile of the *TERT* promoter and *IDH* reflected the survival in different histological sub-types of gliomas. Lowest survival in patients with only *TERT* promoter mutations and longest survival for patients with both *TERT* promoter and *IDH* mutations are most likely indicative of primary glioblastoma and oligodendroglial histologies, respectively, rather than any direct biological effect of the altered genes. Those observations were in accordance with earlier reports [24, 25, 27]. Accordingly, *TERT* promoter mutations did associate strongly with poor survival in the overall analysis of high grade tumors that included primary glioblastomas, high grade astrocytoma and oligodendrogliomas [27, 33]. As shown earlier we observed poor survival in patients with high grade gliomas that had only *TERT* promoter mutations [24, 39]. However, in contrast to earlier reports in this study we did not observe any association between *TERT* promoter mutations and overall survival in patients with primary gliobastoms [40, 41]. The overwhelming occurrence of the *TERT* promoter mutations and in general poor survival associated with primary glioblastoma probably precludes such an association [1, 5].

An earlier study showed the effect of *TERT* promoter mutations on poor survival in primary gliobastoma patients that did not receive chemotherapy [40]. In this study we observed that patients with high grade tumors that did not receive treatment survived least. However, the differences in survival with and without *TERT* promoter mutations was significant only in those patients that were treated with adjuvant therapy together with alkylating agents either temozolomide or dacarbazine.

In astrocytomas, survival was primarily defined by *IDH* mutations and 1p/19q deletions. Patients with these alterations associated with significantly beneficial prognosis; however, *TERT* promoter mutations in the absence of the 1p/19 deletions, though rare, showed debilitating effect on patient survival. In general patients with oligoastrocytomas and oligodendrogliomas followed a similar pattern of survival as astrocytic patients; however, a near absence of *TERT* promoter mutations without *IDH* mutations precluded an independent assessment of the effect of the former alterations as has been the case in earlier studies [24]. The same trend was also discernible for all low grades tumors put together; nevertheless a multivariate model for progression free survival did show association of worst prognosis with *TERT* promoter mutations and best prognosis for patients with 1p/19q deletions.

The *TERT* promoter mutations occur mainly at –124 bp and –146 bp positions from ATG start site of *TERT* promoter. Those positions correspond to the genomic coordinates of Chr5:1, 295, 228 (hg19 co-ordinate) and Chr5:1, 295, 250 (hg19 co-ordinate), which led to the erroneous labeling of the alterations as C228T and C250T [42, 43]. Although all reported mutations in the *TERT* promoter including the causal –57 C > A germline single nucleotide change reported in a melanoma family are joined by a common feature of de novo creation of CCGGAA/T sequence, which, besides being a recognition motif for Ets transcription factors, is a specific binding motif for ternary complex factors like Elk1 and Elk4 [23]. The mutations as presumed resulted in increased promoter activity and have been shown as the main mechanism for *TERT* up-regulation, which conceivably would lead to increased telomerase levels [32, 44, 45]. With the exception of melanoma where the –146C > T mutation is more frequent than any other base change in the *TERT* promoter, in most other malignancies –124 C > T has been reported to be the most common alteration [28]. In this study we observed that while tumors with any of the two mutations showed significantly increased *TERT* transcription, the increase in the expression in tumors with the –124 C > T mutation was significantly higher than in tumors with the –146 C > T mutations. One of the plausible reasons for the novel observation of differences in *TERT* transcription due to two mutations with apparently same phenotype could be context specific variations in accessibility to transcription factors through chromatin remodeling.

The promoter mutations not only affected *TERT* transcription, we also for the first time in glioma observed influence on relative telomere lengths. The tumors with *TERT* promoter mutations in accordance with earlier observations in thyroid cancer had shorter telomeres than tumors without the mutations [44]. Presumed acquisition of the somatic *TERT* promoter mutations at the point of telomere crisis in the course of cellular transformation has been cited as a reason for an association with telomere length. The critically shortened telomeres in oncogene-driven proliferating tumor cells, in the absence of active telomerase, are thought to be causal in cell cycle arrest through activation of signaling cascade involving check point inhibitors [46, 47]. Bypass of replicative senescence and continued telomere attrition in affected cells are postulated to be causal for unstable genomes through cycles of end-fusions and breakages [48]. Selection pressure, leading to acquisition of *TERT* promoter mutations and regeneration of telomerase to overcome telomeric crisis, probably provides the context for shorter telomeres in tumors with mutations [46].

Tumors with *IDH* mutations in the absence of *TERT* promoter mutations had telomere lengths similar to those without mutations. Biologically *IDH* mutations are known to deregulate metabolic process. Mutant *IDH* proteins prevent normal conversion of isocitrate and lead to formation of the R-enantiomer of 2-hydroxylglutarate (R-2-HG) instead of a-ketoglutarate (a-KG) [49]. Increased levels of R-2-HG, besides effecting accumulation of hypoxia-inducible factor 1a, also alters epigenetic modification of histone and DNA methylation through inhibition of a-KG dependent dioxygenases [50]. While *TERT* promoter mutations independently associate with poor in survival in many cancers, the mutations also associate with reduced telomere length. Here we show that *IDH* mutations that are in general markers of better prognosis in glioma do not associate with changed telomere length in tumors.

An inverse correlation between relative telomere length and TERT expression in this study was in accorandance with an earlier report that showed shorter telomere length in gliobastoma that were positive for TERT expression and telomerase activity than those glioblastoma that were negative for both [51]. Conversely, the inverse correlation between the two also supported the observation of shorter telomeres in tumors with *TERT* promoter mutations than without mutations as an increased TERT expression is associated with the presence of mutations.

Development of glioma subtypes along discrete pathways is characterized by the different genetic alterations that emerge along the way [3, 5, 13, 24, 52]. *TERT* promoter mutations add further complexity to the scenario but at the same time hold clear potential as prognostic markers. A combination of *TERT* promoter and *IDH* mutations support placement of gliomas into the subgroups, which is also reflected in survival and can be of considerable use in making appropriate treatment decisions. Data suggest that patients with *TERT* promoter mutations in tumors probably require more aggressive treatment than their wildtype counterparts. Further studies will help in elucidating the value of *TERT* promoter mutations as biomarkers in clinical practice and eventual therapeutic targets. Expression data and an association with shorter telomeres already strongly indicate the role of the *TERT* promoter mutations not only in glioma, but many other cancer types, and future functional studies will aid placing the *TERT* promoter mutations into the right context.

## METHODS

### Patients and tumor samples

Informed consent was obtained from all patients included in the study. The study was approved by the appropriate ethical committee. 303 primary tumors and additional 22 recurrences were collected between March 2010 and August 2013. Fresh tumor tissue was obtained directly from the operating theatre, snap-frozen and processed for further analysis as described below. In the majority of cases tumor samples were taken based on the MRI image using neuronavigation. Neuropathological diagnosis was made from paraffin-embedded tissue by a board-certified neuropathologist. Clinical data was collected in a database which included parameters such as patient age at first diagnosis, sex, histology at primary diagnosis and at recurrence, progression-free survival and overall survival or date of last patient contact. Progression-free survival was assessed on MRI imaging according to Macdonald criteria. Follow-up data was obtained from the family physicians and from the German Cancer Registry. The patients were treated according to the appropriate guidelines or in clinical trials. The date and number of surgeries, type and number of radiation and chemotherapy were documented.

### DNA and RNA extraction

From fresh frozen glioma samples, DNA and RNA were extracted using the QIAGEN AllPrep DNA/RNA MiniKit. Tissues were homogenized in a Tissuelyser LT (Qiagen, Hilden, Germany) with 5 mm stainless steel beads in 600 ml RLT buffer and were further processed. Concentrations of total DNA and RNA in all samples were measured using an ultraviolet–visible spectrophotometer (NanoDrop Technologies, Wilmington, USA) and absorption ratio at 260/280 nm was determined. RNA consistency was examined for a representative number of samples using the Bioanalyzer 2100 System (Agilent Technologies, Palo Alto, CA) with the corresponding RNA nanochips.

### Mutational analysis by sequencing

Mutations in the *TERT* promoter region (from position −27 to −286 from ATG start site) were identified by PCR and Sanger sequencing. Mutational status of *IDH1* was cross-validated by sequencing for all available samples. Mutations in *IDH2* were confirmed by sequencing if the respective probes indicated a signal in MLPA. PCR was carried out in a 10 μl volume containing 10 ng DNA, 50 mM KCl, 0.11 mM dNTP and 0.11 mM of each primer. Concentrations of MgCl_2_ and other additives and temperature conditions were adjusted according to the primer sequences (Supplementary Table 11). Amplified products were purified with Exosap (GE Healthcare, Buckinghamshire, UK) to remove unused primer and were subjected to 35 cycles of sequencing reaction with a di-deoxy terminator kit and forward and reverse primers in separate reactions (BigDyeTerminator v3.1 Cycle Sequencing Kit, Applied Biosystems, Austin, TX, USA). Sequencing reaction products were precipitated with ethanol and analyzed on a capillary sequencer (AbiPrism 3130xl Genetic Analyzer). The sequencing data were analyzed using Geneious Pro 5.6.5 software with reference to the sequences from the NCBI gene database, *TERT* (chr5: 1, 295, 071–1, 295, 521), *IDH1* (NC_000002.12) and *IDH2* (NC_000015.).

### Multiplex ligation-dependent probe amplification (MLPA)

To detect deletions at various relevant sites we used the P088-C1 probemix (SALSA MLPA P088 Oligodendroglioma probemix, MRC-Holland, The Netherlands). This probemix can be used to detect loss of chromosome arms 1p and 19q, as well as copy number changes in CDKN2A and CDKN2B genes. Furthermore, it contains mutation-specific probes for IDH1 (R132H and R132C) and IDH2 (R172K and R172M). In addition, 14 reference probes are included, detecting different chromosomal locations known to be silent in glioma. Since probes for hot spot mutations in IDH1 (R132H and R132C) and IDH2 (R172K and R172M) were not established for the use in tumor tissue by the supplier, results were cross-validated by Sanger sequencing and we can confirm consistent results for both methods. Fifteen glioma samples were analyzed with a second kit also covering the 9p21 locus to compare results (SALSA MLPA ME024 9p21 CDKN2A/2B; MRC Holland, The Netherlands). The method was performed following the suggested protocol. In short, 50 ng of DNA per sample were subjected to 16 h of incubation with the probe mix and afterwards processed in a ligation reaction, followed by a multiplex PCR. Fragment analysis was performed on a capillary sequencer (AbiPrism 3130xl Genetic Analyzer). The results were evaluated using Coffalyser software (MRC-Holland); threshold to define deletions was set at the suggested delta value of 0.3.

### Measurement of TERT mRNA expression

For measurement of *TERT* expression, reverse transcription reaction was performed using between 0.75 and 1.0 mg RNA and random hexamer primers using a cDNA synthesis kit (Thermo Scientific, Waltham, USA). *TERT* expression levels were then determined by quantitative real-time PCR using a Sybr Green kit (Qiagen). The real-time PCR was carried out in triplicates on a 384-well layout using primers specific for *TERT* (Supplementary Table 11) and primers for the *GUSB* gene (Qiagen), a housekeeping gene used as an internal standard. *TERT* expression levels were calculated using GUSB expression as a reference and relative quantification was performed using the ΔΔC_T_ method and log2 transformation.

### Measurement of telomere length

Relative telomere length in tumor DNA was measured using the monochrome multiplex PCR assay as described previously including minor modifications [53, 54]. Described briefly, reactions were performed in triplicates in an optical 384-well reaction plate in a 10 μl reaction volume using 2 μl of 5X HOT FIREPol Probe qPCR Mix Plus with ROX (Solis BioDyne), 1.5 μM of Syto 9 (Invitrogen) and 5–10 ng of genomic DNA. Four primers (Supplementary Table 11) were used in each reaction to amplify telomere DNA (telg at 200 nM and telc at 400 nM) and the albumin gene (albugcr2 at 200 nM and albdgcr2 at 400 nM). Real-time PCR experiments were performed on an Applied Biosystems Viia-7 instrument using two simultaneous programs to acquire the respective CT values for telomere sequences and the albumin gene. The conditions for telomere sequence amplification were 95°C/15 min, 2 cycles of 95°C/20 sec and 49°C/1 min, followed by 25 cycles of 85°C/20 sec with signal acquisition at 59°C/30 sec. The conditions for albumin gene were 35 cycles of 95°C/15 sec, 85°C/30 sec, with signal acquisition at 84°C/30 sec. The specificity of all amplifications was determined by melting curve analysis done at default settings (95°C/15 sec, 60°C/1 min with continuous signal acquisition at 0.05°C/sec ramping, 95°C/15 sec). Seven concentrations of a reference DNA sample (genomic DNA pooled from 10 healthy individuals) were included in triplicates in a 2-fold serial dilution (from 20 ng to 0.3 ng) to generate standard curves for telomere (T) and albumin (S) PCR products, respectively. The quality control was performed using the Applied Biosystems Viia-7 software. The standard curve was used to quantify the telomere and albumin genes based on the respective CT values and the obtained triplicate values were averaged. The relative telomere lengths was expressed as the ratio between T/S. Inter-assay variation and intra-assay variation was determined by duplicating the reference DNA for all the dilutions in all the assays performed.

### Statistical analysis

Associations between the *TERT* promoter mutations and other parameters were determined by chi^2^-test. A difference was considered statistically significant if the *P*-value was 0.05 or smaller. Odds ratios were calculated to assess the effect size. Overall survival was studied using univariate and multivariate Cox proportional hazards modeling, where appropriate. Survival endpoints were analyzed by Kaplan-Meier estimates and differences between the curves were analyzed with log-rank test.

## SUPPLEMENTARY FIGURES AND TABLES


