# Supplementary material for: TERT promoter mutations and telomere length in adult malignant gliomas and recurrences
Source: Oncotarget. 2015 Mar 12;6(12):10617–33. doi: 10.18632/oncotarget.3329 (PMC4496380; doi:10.18632/oncotarget.3329)
Supplement: Supplementary file 1 [file oncotarget-06-10617-s001.pdf]

SUPPLEMENTARY FIGURES AND TABLES

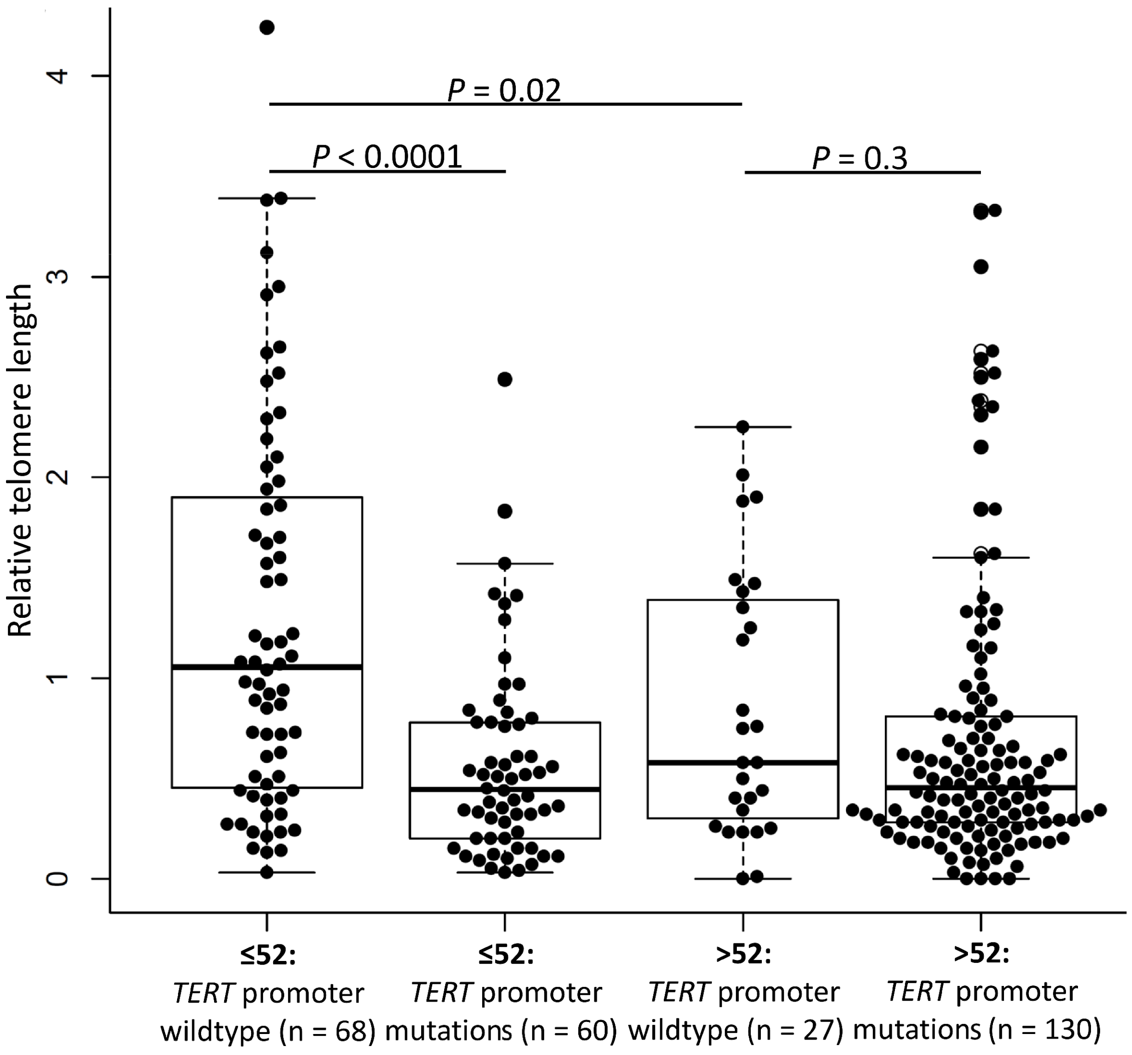

Supplementary Figure 1: Telomere lengths in glioma tumors stratified according to age. Relative telomere length in gliomas without and with *TERT* promoter mutations, stratified according to median age of the patients. Experiments were carried out in triplicate and box plots represent mean ± s.e.m. *P*-values were determined by *t*-test.

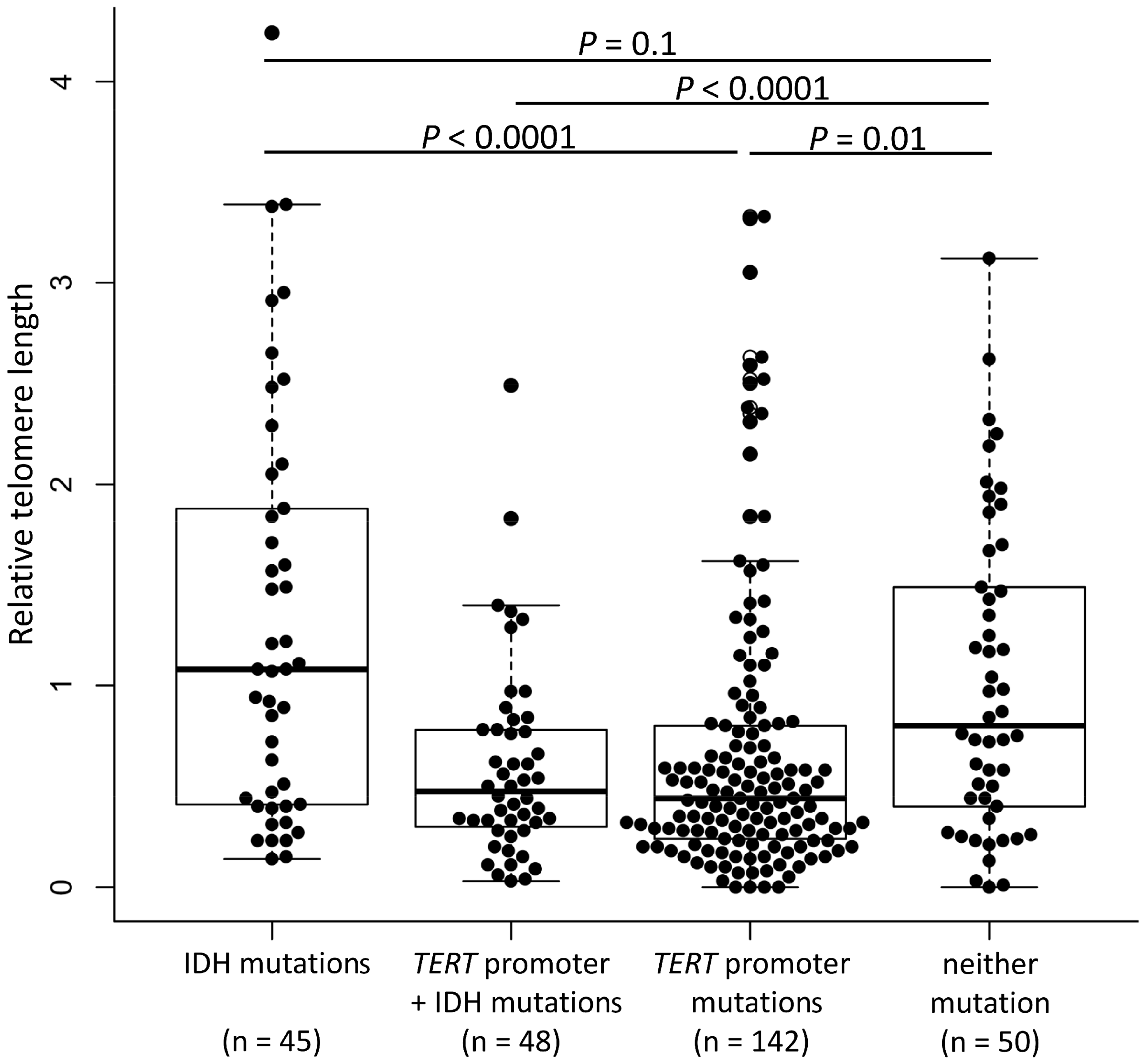

Supplementary Figure 2: Relative telomere lengths in glioma samples according to mutation status of IDH and the *TERT* promoter. Comparison of relative telomere lengths in gliomas with and without mutations in *IDH* and the *TERT* promoter, respectively. Experiments were carried out in triplicate and box plots represent mean ± s.e.m. *P*-values were determined by *t*-test.

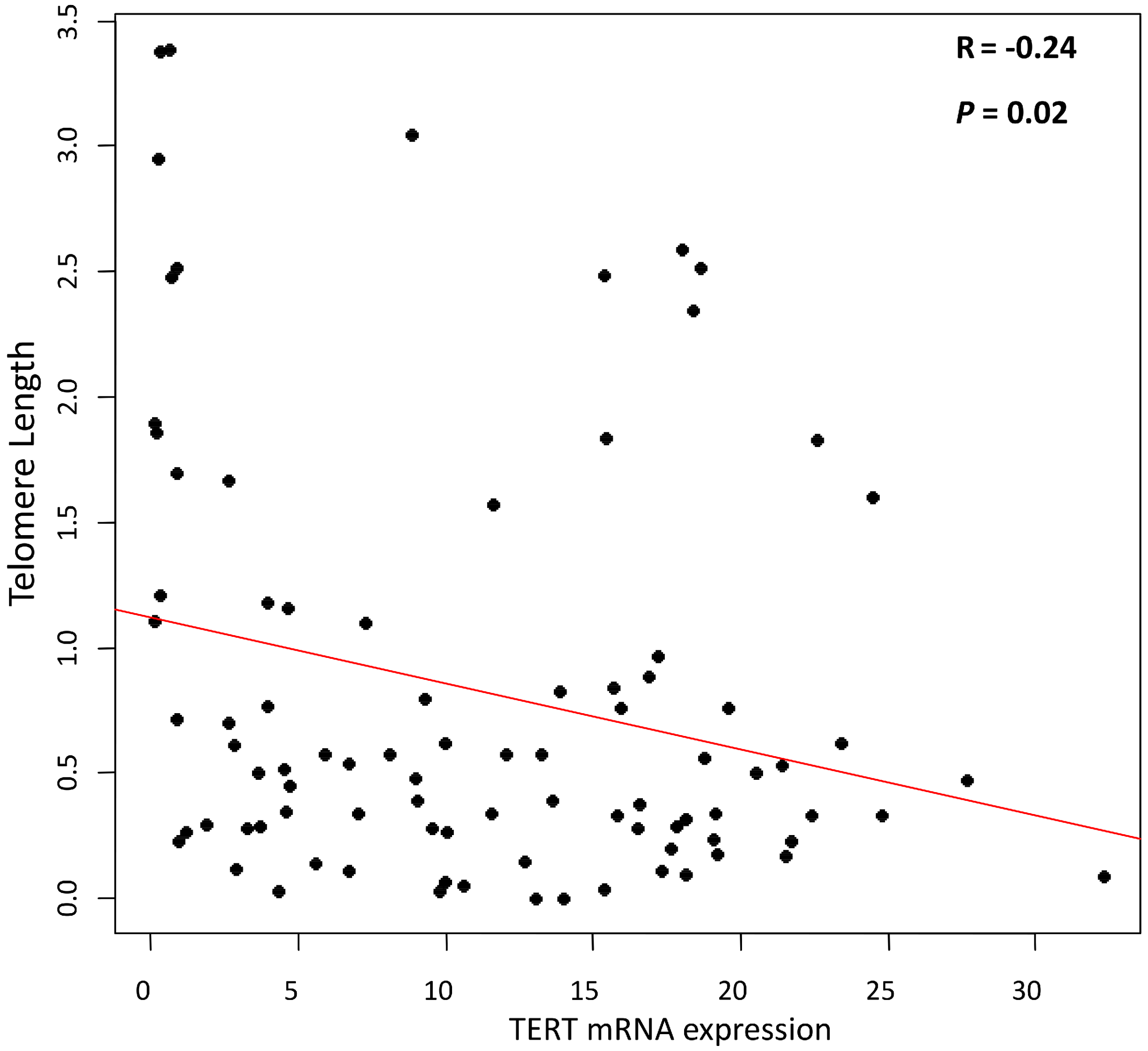

Supplementary Figure 3: Correlation between telomere length and TERT mRNA expression. Investigation of correlation using linear regression between relative telomere length and relative TERT mRNA expression for tumors were both values were available (n = 88). P-values were determined by ANOVA.

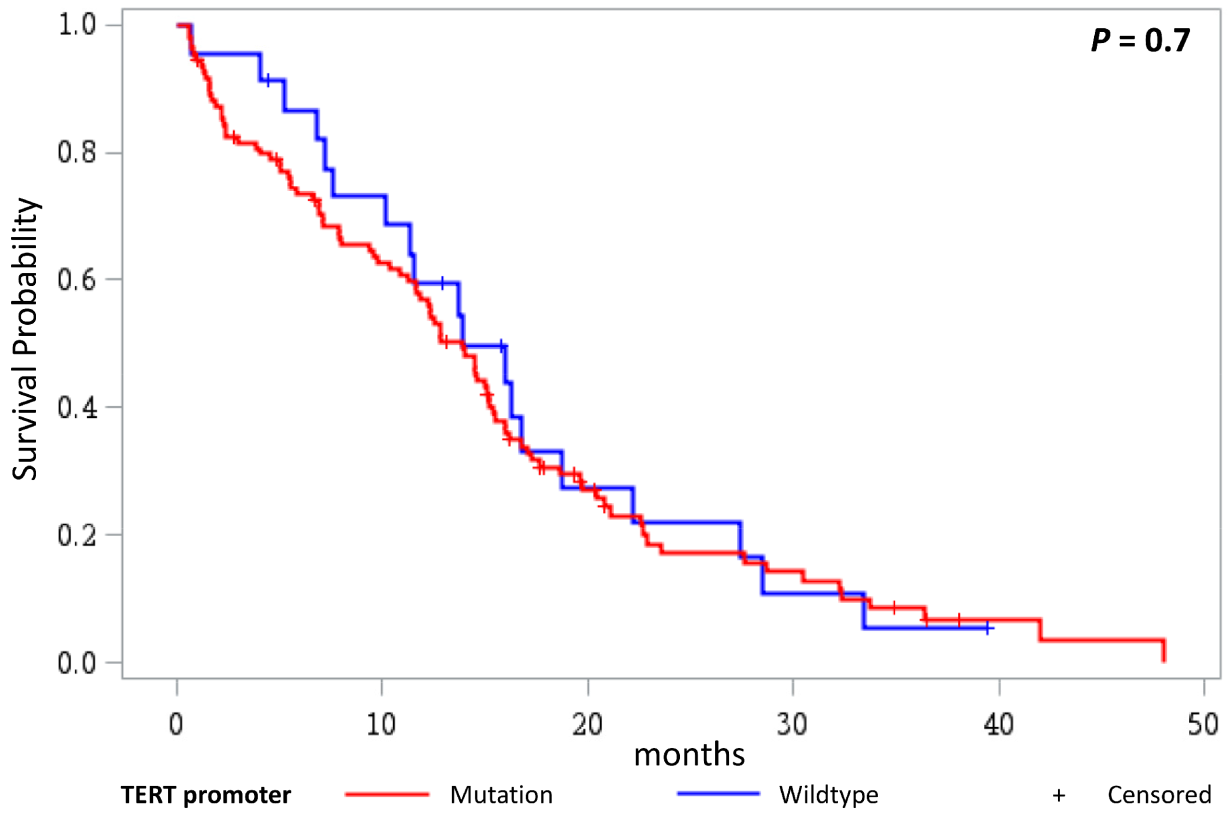

	Total	Failed	censored
TERTmut	111	91	20
TERTwt	23	19	4
**Total**	134	110	24

Supplementary Figure 4: Overall survival in patients with primary glioblastoma according to *TERT* promoter status.

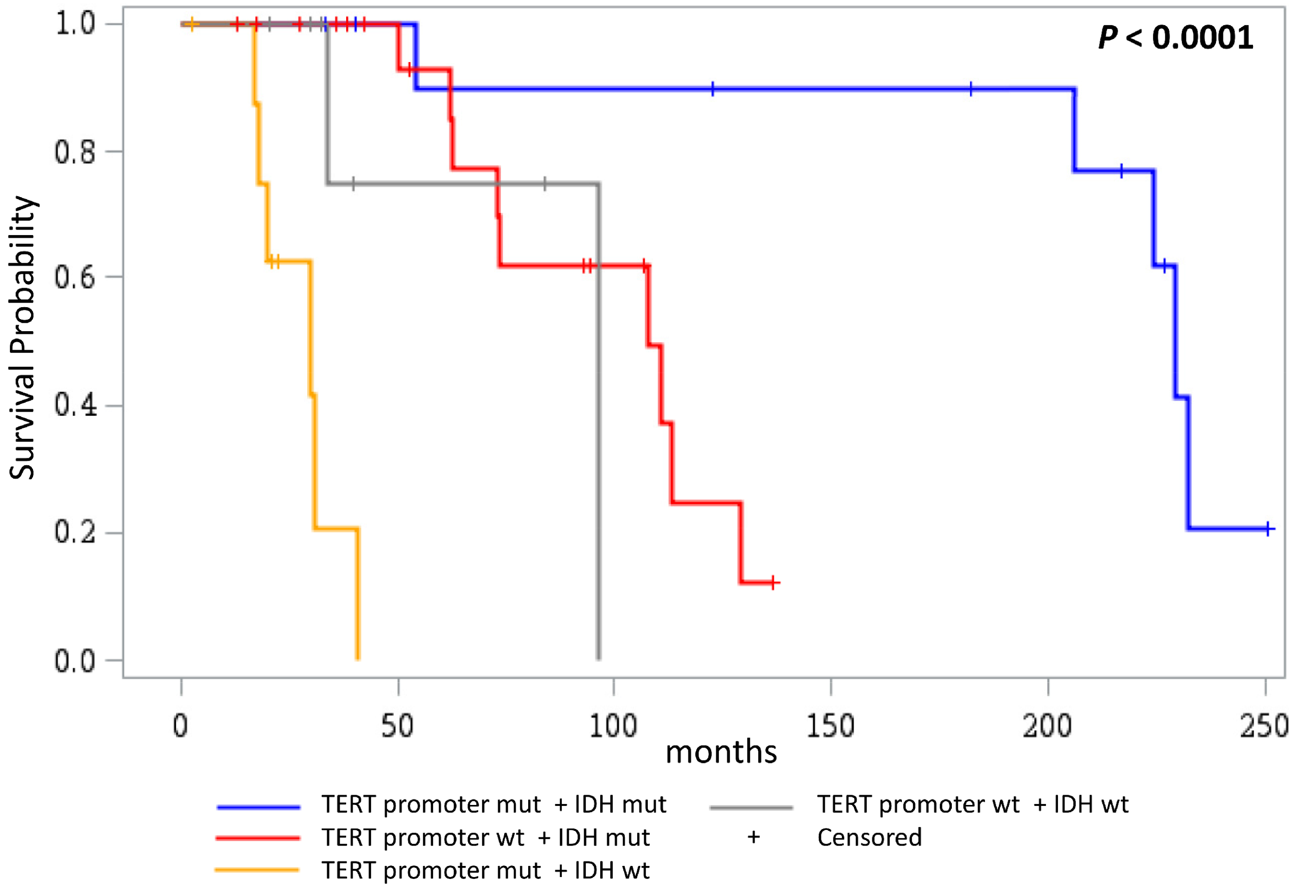

	Total	Failed	censored
TERTmut + IDHmut	13	5	8
TERTwt + IDHmut	21	9	12
TERTmut + IDHwt	9	6	3
TERTwt + IDHwt	7	2	5
**Total**	50	22	28

Supplementary Figure 5: Overall survival according to combined status of *TERT* promoter and *IDH* mutation in patients with astrocytoma.

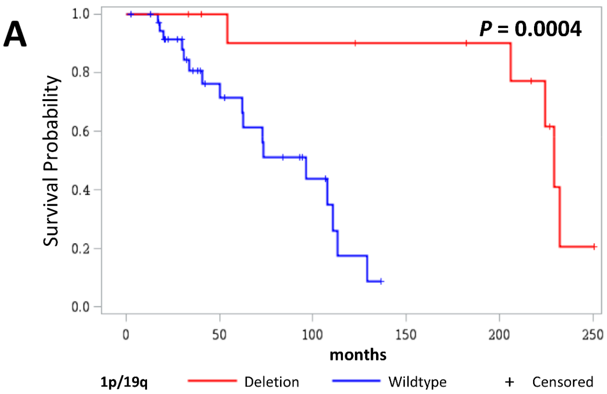

1p/19q	Total	Failed	censored
wt	38	17	21
deletion	12	5	7
**Total**	50	22	28

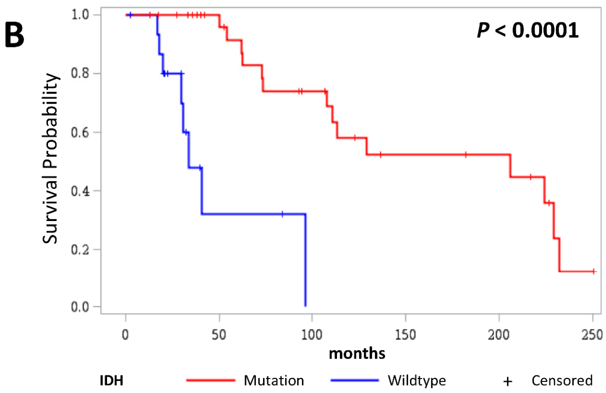

IDH	Total	Failed	censored
wt	16	8	8
mut	34	14	20
**Total**	50	22	28

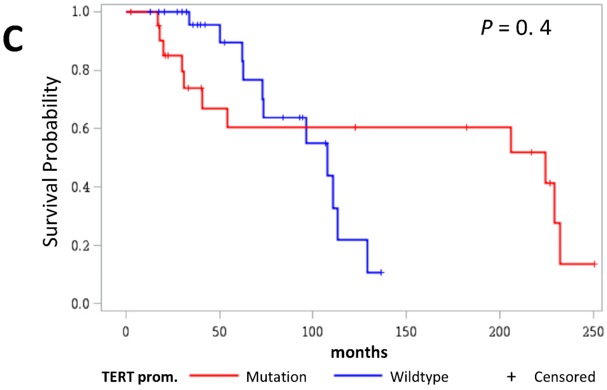

TERT	Total	Failed	censored
wt	28	11	17
mut	22	11	11
**Total**	50	22	28

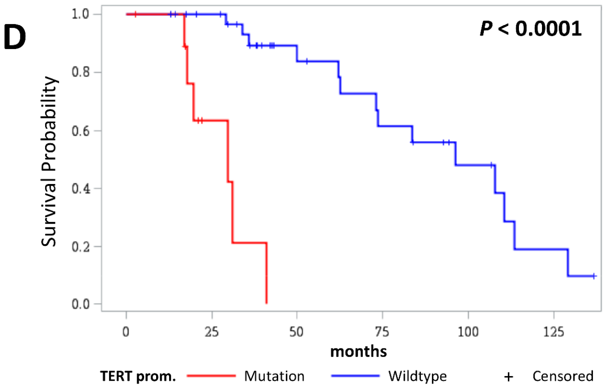

TERT *	Total	Failed	censored
wt	28	11	17
mut	10	6	4
**Total**	38	17	21

Supplementary Figure 6: Overall survival in patients with astrocytoma according to alterations in the *TERT* promoter, *IDH* and at 1p/19q. Kaplan Meier analysis of differences in overall survival in astrocytoma patients stratified according to **(A)** co-deletions in 1p/19q, **(B)** *IDH* mutations and **(C)** *TERT* promoter mutations. **(D)** To assess the effect of *TERT* promoter mutations without the influence of 1p/19q co-deletions, patients that carried the co-deletions were removed from the analysis and survival analysis stratified by *TERT* promoter mutations was repeated. *Analysis of survival of patients with *TERT* promoter mutations and without 1p/19q co-deletions.

A

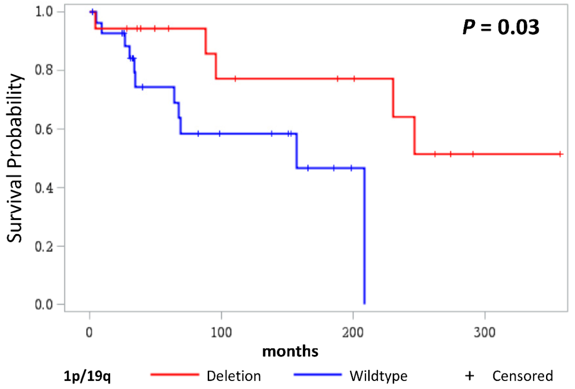

1p/19q	Total	Failed	censored
wt	28	11	17
deletion	17	5	12
**Total**	45	16	29

B

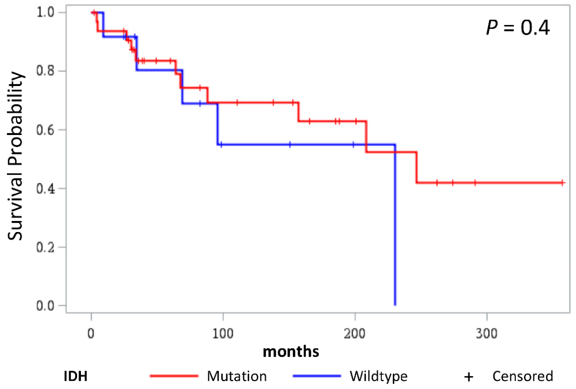

IDH	Total	Failed	censored
wt	12	5	7
mut	33	11	22
**Total**	45	16	29

C

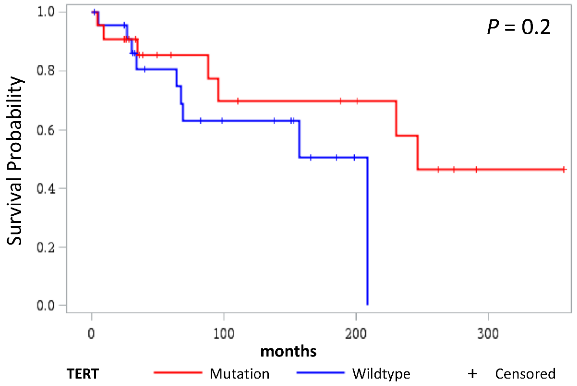

TERT	Total	Failed	censored
wt	23	9	14
mut	22	7	15
**Total**	45	16	29

D

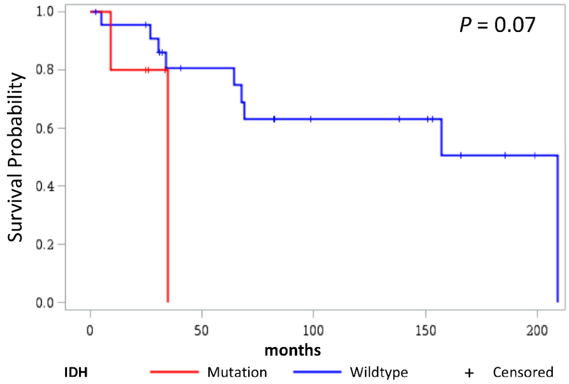

TERT *	Total	Failed	censored
wt	23	9	14
mut	5	2	3
**Total**	28	11	17

Supplementary Figure 7: Overall survival in patients with oligoastrocytoma according to alterations in the *TERT* promoter, *IDH* and at 1p/19q. Kaplan Meier analysis of differences in overall survival in patients with oligoastrocytoma stratified according to **(A)** co-deletions in 1p/19q, **(B)** *IDH* mutations and **(C)** *TERT* promoter mutations. **(D)** To assess the effect of *TERT* promoter mutations without the influence of 1p/19q co-deletions, patients that carried the co-deletions were removed from the analysis and survival analysis stratified by *TERT* promoter mutations was repeated. *Analysis of survival of patients with *TERT* promoter mutations and without 1p/19q co-deletions.

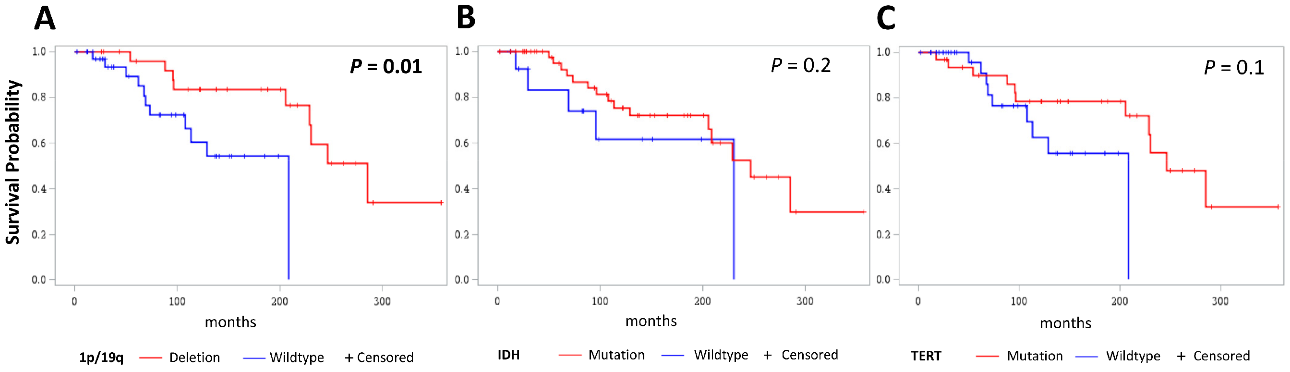

1p/19q	Total	Failed	censored
wt	36	11	25
deletion	28	9	19
**Total**	64	20	44

IDH	Total	Failed	censored
wt	14	5	9
mut	50	15	35
**Total**	64	20	44

TERT	Total	Failed	censored
wt	33	9	24
mut	31	11	20
**Total**	64	20	44

Supplementary Figure 8: Overall survival in patients with low grade gliomas according to alterations in the *TERT* promoter, *IDH* and at 1p/19q. Kaplan Meier analysis of differences in overall survival in patients with low grade gliomas (grade II), including astrocytomas, oligoastrocytomas and oligodendrogliomas. For survival analysis patients were stratified according to **(A)** co-deletions in 1p/19q, **(B)** *IDH* mutations and **(C)** *TERT* promoter mutations. In **(C)** *TERT* promoter mutations seem to confer a better survival, however, this association is due to co-occurrence with 1p/19q co-deletions. In low grade gliomas only three patients harboured *TERT* promoter mutations without 1p/19q co-deletions in their tumors.

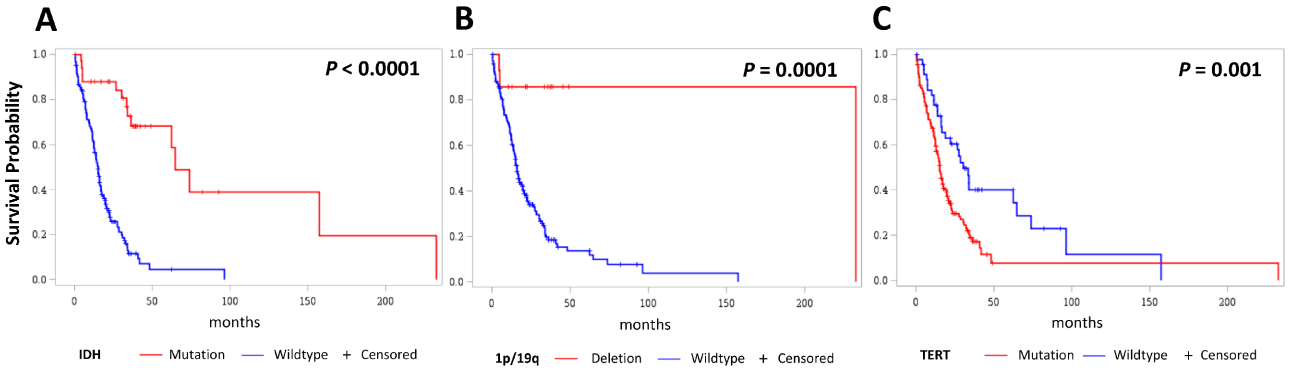

IDH	Total	Failed	censored
wt	151	116	35
mut	34	14	20
**Total**	185	130	55

1p/19q	Total	Failed	censored
wt	171	127	44
deletion	14	3	11
**Total**	185	130	55

TERT	Total	Failed	censored
wt	139	101	38
mut	46	29	17
**Total**	185	130	55

Supplementary Figure 9: Overall survival in patients with high grade gliomas according to alterations in the *TERT* promoter, *IDH* and at 1p/19q.

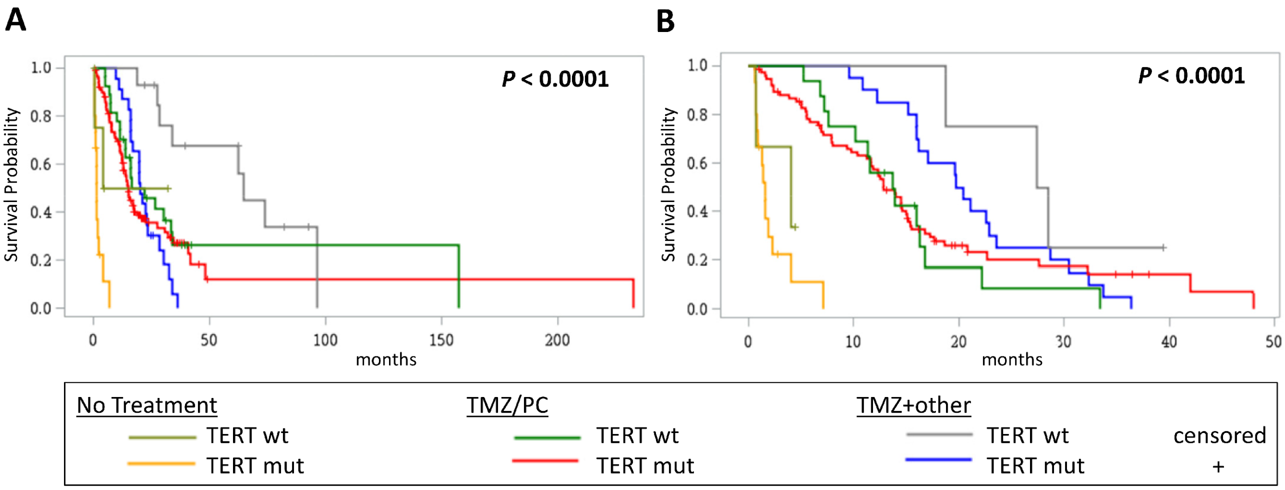

TERT	Treatment Group	Total	Failed	censored
mut	none (1)	15	13	2
mut	TMZ/PC (2)	101	67	34
mut	TMZ+other (3)	23	21	2
wt	none (1)	5	2	3
wt	TMZ/PC (2)	27	19	8
wt	TMZ+other (3)	14	8	6
**Total**		185	130	55

TERT	Treatment Group	Total	Failed	censored
mut	none (1)	15	13	2
mut	TMZ/PC (2)	76	58	18
mut	TMZ+other (3)	20	20	0
wt	none (1)	3	2	1
wt	TMZ/PC (2)	16	14	2
wt	TMZ+other (3)	4	3	1
**Total**		134	110	24

Supplementary Figure 10: Overall survival in glioma patients according to different treatment regimens. Kaplan Meier analysis of overall survival in glioma patients receiving different treatment regimens and additionally stratified according to their *TERT* promoter mutational status. Patients were stratified into three groups, where group 1) did not receive any treatment, group 2) received radiotherapy alone or in combination with PC or TMZ and group 3) received TMZ and radiotherapy combined with a third adjuvant. Survival analysis then was performed according to the different treatment groups and the TERT promoter mutational status in **(A)** the complete set of glioma patients and **(B)** patients with primary glioblastomas.

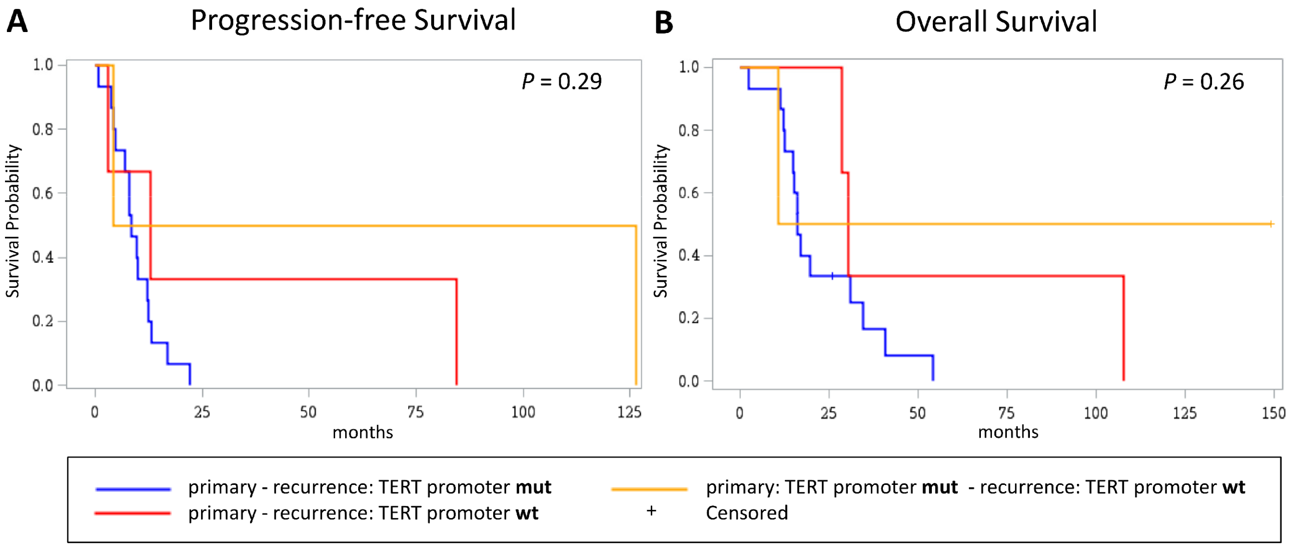

	TERT promoter	Total	Failed	censored
Primary - recurrence	mut/mut	15	15	0
Primary - recurrence	wt/wt	3	3	0
Primary - recurrence	mut/wt	2	2	0
**Total**		20	20	0

	TERT promoter	Total	Failed	censored
Primary - recurrence	mut/mut	15	14	1
Primary - recurrence	wt/wt	3	3	0
Primary - recurrence	mut/wt	2	1	1
**Total**		20	18	2

Supplementary Figure 11: Survival analysis in patients with primary tumors and recurrences. Analysis of **(A)** progression-free and **(B)** overall survival in patients for whom information about their primary glioma tumor and corresponding recurrence was available. Patients were grouped according to *TERT* promoter mutations, where both primary and recurrence carried *TERT* promoter mutations or both lesions did not carry the mutations. A third group consisted of the two patients that showed *TERT* promoter mutations in their primary tumors but were wildtype in corresponding recurrences.

Supplementary Table 1: Distribution of clinical parameters and mutations in different histological classifications

	All	Astrocytomas	Oligoastrocytomas	Oligodendrogliomas	Primary Glioblastomas
	n = 303	*n* = 56	*n* = 55	*n* = 27	*n* = 165
Gender					
male	192	38	34	13	107
female	111	18	21	14	58
Age					
≤ 55	152	48	47	19	38
> 55	151	8	8	8	127
Grade					
low (II)	78	33	34	11	0
high (III + IV)	225	23	21	16	165
IDH					
wildtype	203	19	16	11	157
mutation	100	37	39	16	8
9p21					
wildtype	138	30	34	16	58
mutation	119	17	13	9	80
missing	*46*	*9*	*8*	*2*	*27*
Deletions at 1p and/or 19q					
wildtype	185	34	23	11	117
deletion	74	15	24	14	21
missing	*44*	*7*	*8*	*2*	*27*
1p/19q codeletions					
wildtype	214	39	28	11	136
codeletion	45	10	19	14	2
missing	*44*	*7*	*8*	*2*	*27*

Supplementary Table 2: Association of *TERT* promoter mutations with mutations in IDH1/2 and deletions at 9p21 and 1p19q in different glioma subtypes

	Astrocytomas		Oligoastrocytomas		Oligodendrogliomas		Primary Glioblastomas	
	***n* = 56**		***n* = 55**		***n* = 27**		***n* = 165**	
	***TERT* promoter**		***TERT* promoter**		***TERT* promoter**		***TERT* promoter**	
	**wildtype**	**mutation**	**wildtype**	**mutation**	**wildtype**	**mutation**	**wildtype**	**mutation**
IDH<br>wildtype<br>mutation	10<br>24	9<br>13	8<br>21	8<br>18	7<br>1	4<br>15	28<br>5	129<br>3
	OR = 0.60; 95% CI 0.20 – 1.85; *P* = 0.37		OR = 0.86; 95% CI 0.27 – 2.75; *P* = 0.8		OR = 26.25; 95% CI 2.46 – 280.20; ***P* = 0.001**		OR = 0.13; 95% CI 0.03 – 0.58; ***P* = 0.002**	
**9p21**<br>Wildtype<br>mutation	21<br>7	9<br>10	18<br>5	16<br>8	5<br>2	11<br>7	16<br>10	42<br>70
	OR = 3.33; 95% CI 0.96 – 11.54; ***P* = 0.05**		OR = 1.80; 95% CI 0.49 – 6.64; *P* = 0.4		OR = 1.60; 95% CI 0.24 – 10.57; *P* = 0.6		OR = 2.67; 95% CI 1.11 – 6.42; ***P* = 0.03**	
**1p and/or 19q**<br>Wildtype<br>deletion	26<br>3	8<br>12	18<br>5	5<br>19	7<br>0	4<br>14	22<br>4	95<br>17
	OR = 13.0; 95% CI 2.92 – 57.85; ***P* = 0.0002**		OR = 13.70; 95% CI 3.38 – 55.32; ***P* < 0.0001**		OR = 4.50; 95% CI 1.90 – 10.68; ***P* = 0.0004**		OR = 0.98; 95% CI 0.30 – 3.22; *P* = 1.0	
**1p/19q**<br>wildtype<br>codeletion	29<br>0	10<br>10	23<br>0	5<br>19	7<br>0	4<br>14	26<br>110	0<br>2
	OR = 2.00; 95% CI 1.29 –3.10; *P* < 0.0001		OR = 4.80; 95% CI 2.20 – 10.47; ***P* < 0.0001**		OR = 4.50; 95% CI 1.90 – 10.68; ***P* = 0.0004**		OR = 1.2; 95% CI 1.0 – 1.04; *P* = 0.5	

P –values were derived from χ^2-test and considered statistically significant if < 0.05.
Bold font indicates statistical significance.

Supplementary Table 3: Multivariate Model in complete set of Gliomas grouped according to *TERT* promoter and *IDH* status

Parameter		*P*	HR	95% CI	
Group	*TERT+IDH*	**0.03**	**0.22**	**0.08**	**0.59**
Group	*IDH*	0.72	1.14	0.56	2.36
Group	*TERT*	0.78	0.93	0.55	1.56
Age		**< .0001**	**1.04**	**1.02**	**1.06**
Grade	III	**< .0001**	**5.22**	**2.41**	**11.33**
Grade	IV	**< .0001**	**32.92**	**12.89**	**84.04**
CDKN2A/B	Deletion	0.43	1.15	0.81	1.64
Treatment - Group	PC	0.23	1.79	0.7	4.61
Treatment - Group	RT	0.06	1.75	0.99	3.12
Treatment - Group	TMZ+ot	0.72	0.93	0.61	1.41
Treatment - Group	none	**< .0001**	**5.61**	**2.94**	**10.72**

Supplementary Table 4: Multivariate Model in Astrocytomas grouped according to *TERT* promoter and *IDH* status

Parameter		*P*	HR	95% CI	
group	*TERT+IDH*	0.02	0.02	0.001	0.54
group	*IDH*	0.42	0.35	0.03	4.33
group	*TERT*	**0.02**	**37.67**	**1.63**	**870.82**
Age		0.11	1.06	1.00	1.13
Grade	III	0.23	0.35	0.07	1.91
Grade	IV	0.58	0.40	0.01	10.89
CDKN2A/B	Deletion	**0.01**	**6.11**	**1.47**	**25.37**
Treatment - Group	PC	1.00	0.00	0.00	-
Treatment - Group	RT	0.24	7.38	0.27	201.46
Treatment - Group	TMZ+ot	0.25	2.69	0.50	14.47
Treatment - Group	none	0.99	0.00	0.00	-

Supplementary Table 5: Multivariate Model in Astrocytomas

Parameter		*P*	HR	95% CI	
TERT promoter	Mutation	**0.02**	**37.65**	**1.63**	**869.91**
IDH	Mutation	0.42	0.35	0.03	4.32
1p/19q co-deletion	Deletion	**0.002**	**0.002**	**0.00**	**0.09**
CDKN2A/B	Deletion	**0.01**	**6.12**	**1.48**	**25.37**
Age		0.11	1.06	0.99	1.13
Grade	III	0.22	0.35	0.07	1.90
Grade	IV	0.58	0.40	0.01	10.88
Treatment – Group*	RT	0.24	7.38	0.27	201.53
Treatment – Group*	TMZ+ot	0.25	2.69	0.50	14.48

*excluded from analysis: treatment types: PC (*n* = 1), None (*n* = 7)

Supplementary Table 6: Multivariate Model in Oligoastrocytomas

Parameter		*P*	HR	95% CI	
TERT promoter	Mutation	0.62	2.05	0.12	35.50
IDH	Mutation	0.27	0.16	0.01	3.98
1p/19q co-deletion	Deletion	0.54	0.57	0.09	3.75
CDKN2A/B	Deletion	**0.05**	**0.19**	**0.04**	**1.02**
Age		0.10	1.06	0.99	1.14
Grade	III	**0.02**	**16.45**	**1.64**	**165.01**
Treatment - Group*	PC	0.60	0.68	0.16	2.84
Treatment - Group*	RT	0.33	0.30	0.03	3.46
Treatment - Group*	TMZ+ot	0.20	0.27	0.04	1.96

*excluded from analysis: treatment types: None (*n* = 4)

Supplementary Table 7: Multivariate Model in Oligoastrocytomas (Exclusion of 1p/19q co-deletions)

Parameter		*P*	HR	95% CI	
TERT promoter	Mutation	0.75	1.90	0.04	89.68
IDH	Mutation	0.82	0.67	0.02	19.88
CDKN2A/B	Deletion	0.47	0.50	0.07	3.37
Age		0.24	1.07	0.96	1.19
Grade	III	0.12	11.37	0.53	242.59
Grade	IV	0.32	0.34	0.04	2.88
Treatment - Group	RT	0.79	1.60	0.05	48.49
Treatment - Group	TMZ+ot	0.34	0.40	0.06	2.65

Supplementary Table 8: Multivariate Model in Low Grade Gliomas*

Parameter		*P*	HR	95% CI	
TERT promoter	Mutation	0.03	0.24*	0.07	0.87
IDH	Mutation	0.74	0.79	0.20	3.14
CDKN2A/B	Deletion	0.23	0.20	0.65	6.03
Age		**0.02**	**1.08**	**1.01**	**1.14**
Treatment - Group	PC	0.53	0.60	0.12	2.94
Treatment - Group	RT	0.26	0.26	0.04	1.86
Treatment - Group	TMZ+ot	1.21	1.21	0.28	5.18

**HR could not be calculated when 1p19q was included, resulting in a misleadingly negative HR for TERT promoter mutations*

Supplementary Table 9: Multivariate Model in High Grade Gliomas

Parameter		*P*	HR	95% CI	
TERT promoter	Mutation	0.16	1.49	0.86	2.59
1p/19q co-deletion	Deletion	**0.003**	**0.09**	**0.02**	**0.44**
IDH	Mutation	0.67	0.84	0.37	1.89
CDKN2A/B	Deletion	0.61	0.91	0.62	1.33
Treatment - Group	PC	0.66	0.76	0.23	2.53
Treatment - Group	RT	0.28	1.39	0.76	2.53
Treatment - Group	TMZ+ot	0.22	0.75	0.47	1.19
Treatment - Group	none	**< .0001**	**9.58**	**4.99**	**18.40**
Age		**< .0001**	**1.05**	**1.03**	**1.06**

Supplementary Table 10: Comparison of genetic alterations in primary glioma tumors and matched recurrences

Primary tumor / Recurrence	*CDKN2A/B*	1p/19q	*IDH*	*TERT* promoter	rs2853669	Histology at primary diagnosis / Histology final
BT-121	deletion[a]	partial 1p deletion[m]	wt	−124C > T	TT	glioblastoma WHO grade IV
BT-121R1	deletion[a]	partial 1p deletion[m]	wt	−124C > T	TT	glioblastoma WHO grade IV
BT-123	deletion[a]	co-deleltion 1p19q[d]	c.395G > A	−124C > T	CC	oligoastrocytoma WHO grade III
BT-123R1	deletion[a]	co-deleltion 1p19q[d]	c.395G > A	−124C > T	CC	oligoastrocytoma WHO grade III
BT-123R2	deletion[a]	co-deleltion 1p19q[d]	c.395G > A	−124C > T	CC	glioblastoma WHO grade IV
BT-154	wt	wt	wt	−124C > T	TT	glioblastoma WHO grade IV
BT-154R1	wt	wt	wt	−124C > T	TT	glioblastoma WHO grade IV

(*Continued*)

Primary tumor / Recurrence	*CDKN2A/B*	1p/19q	*IDH*	*TERT* promoter	rs2853669	Histology at primary diagnosis / Histology final
BT-159	deletion[a]	wt	wt	−124C > T	TT	oligoastrocytoma WHO grade III
BT-159R1	deletion[a]	wt	wt	−124C > T	TT	oligoastrocytoma WHO grade III
BT-159R2	deletion[a]	wt	wt	−124C > T	TT	glioblastoma WHO grade IV
BT-177	deletion[a]	wt	wt	−124C > T	CT	oligoastrocytoma WHO grade III
BT-177R1	deletion[a]	wt	wt	−124C > T	CT	glioblastoma WHO grade IV
BT-185	wt	wt	wt	wt	TT	glioblastoma WHO grade IV
BT-185R1	wt	wt	wt	wt	TT	glioblastoma WHO grade IV
BT-200	wt	wt	wt	−124C > T	CC	glioblastoma WHO grade IV
BT-200R1	wt	wt	wt	−124C > T	CC	glioblastoma WHO grade IV
BT-215	wt	wt	c.395G > A	wt	CT	anaplastic oligoastrocytoma WHO grade III
BT-215R1	wt	wt	c.395G > A	wt	CT	glioblastoma WHO grade IV
BT-220	wt	wt	c.395G > A	wt	CT	glioblastoma WHO grade IV
BT-220R1	wt	wt	c.395G > A	wt	CT	glioblastoma WHO grade IV
BT-222	deletion[a]	wt	wt	−124C > T	CT	glioblastoma WHO grade IV
BT-222R1	deletion[a]	wt	wt	−124C > T	CT	glioblastoma WHO grade IV
BT-264	wt	wt	wt	−124C > T	CT	glioblastoma WHO grade IV
BT-264R1	wt	wt	wt	−124C > T	CT	glioblastoma WHO grade IV
BT-266	wt	wt	wt	−124C > T	TT	glioblastoma WHO grade IV
BT-266R1 (BT364)	wt	wt	wt	−124C > T	TT	glioblastoma WHO grade IV
BT-43	deletion[a]	wt	wt	−146C > T	TT	glioblastoma WHO grade IV
BT-43R1	deletion[a]	wt	wt	−146C > T	TT	glioblastoma WHO grade IV
BT-62	wt	wt	wt	−146C > T	TT	glioblastoma WHO grade IV
BT-62R1	wt	wt	wt	−146C > T	TT	glioblastoma WHO grade IV
BT-92	deletion[a]	partial 1p deletion[k]	wt	−124C > T	CT	glioblastoma WHO grade IV
BT-92R1	deletion[a]	partial 1p deletion[k]	wt	−124C > T	CT	glioblastoma WHO grade IV
BT-163	deletion[a]	wt	wt	−124C > T	CT	glioblastoma WHO grade IV
BT-163R1	wt	wt	wt	wt	CT	glioblastoma WHO grade IV; necrosis, single tumor cells
BT-171	deletion[a]	wt	c.395G > A	−124C > T	CC	oligodendroglioma WHO grade II
BT-171R1	wt	wt	wt	wt	CC	anaplastic oligoastrocytoma, WHO grade III
BT-261	deletion[a]	wt	wt	−124C > T	CT	glioblastoma WHO grade IV
BT-261R1	wt	wt	wt	−124C > T	CT	glioblastoma WHO grade IV
BT-288	wt	wt	wt	−146C > T	CT	glioblastoma WHO grade IV

(*Continued*)

Primary tumor<br>Recurrence	*CDKN2A/B*	1p/19q	*IDH*	*TERT* promoter	rs2853669	Histology at primary diagnosis<br>Histology final
BT-288R1	wt	partial 19q deletion[h]	wt	−146C > T	CT	glioblastoma WHO grade IV
BT-56	deletion[a]	wt	wt	−124C > T	TT	anaplastic astrocytoma WHO grade III.
BT-56R1	wt	wt	wt	−124C > T	TT	glioblastoma WHO grade IV

[a]comprises MLPA probes for CDKN2A-3, CDKN2A-2a, CDKN2A-1, CDKN2B-2, CDKN2B-1b
[d]comprises MLPA probes for 1 p arm: TNFRSF14–8, TP73–1, PARK7–7, MFN2–10, WNT4–2, PTAFR-4, PRDX1–7, PRDX1–2c, FAF1–4, CDKN2C-1, CDKN2C-3a, PPAP2B-2, MIR101–1–1, FUBP1–8, GTF2B-2, DPYD-1, NRAS-5, NOTCH2–5; 19 q arm: CCNE1–11, PDCD5–1, UPK1A-4, UPK1A-7, TGFB1–3, CIC-10, ZNF296–2, PPP1R15A-2, BAX-4, CHMP2A-4, CHMP2A-2
[h]comprises MLPA probes for TGFB1–3, CIC-10, ZNF296–2, PPP1R15A-2, BAX-4, CHMP2A-4, CHMP2A-2
[k]comprises MLPA probes for PARK7–7, CDKN2C-1, CDKN2C-3a
[m]comprises MLPA probes for PRDX1–7, PRDX1–2c, FAF1–4, CDKN2C-1, CDKN2C-3a

Supplementary Table 11: PCR conditions and primer sequences

Gene	$MgCl_2$ concentration and additives	Size (bp)	Primer sequence	Annealing temperature
TERT promoter: –27 to –286				
forward<br>reverse	2 mM $MgCl_2$;<br>5% Glycerol	260	5′CCCACGTGCGCAGCAGGAC3′<br>5′CTCCCAGTGGATTCGCGGGC3′	60°C
IDH1				
forward<br>reverse	1.5 mM $MgCl_2$;<br>5% Glycerol	286	5′TGGCGTCAAATGTGCCACTATC3′<br>5′ACACATACAAGTTGGAAATTT CTGGGC3′	53°C
IDH2				
forward<br>reverse	1.5 mM $MgCl_2$;<br>5% Glycerol	373	5′AGGACCCCCGTCTGGCTGTG3′<br>5′CGGCCCGGTCTGCCACAAAG3′	61°C
**TERT mRNA expression**				
forward (TERT exon 3)<br>reverse (TERT exon 4)	2.5 mM $MgCl_2$	145	5′CGGAAGAGTGTCTGGAGCAA3′<br>5′GGATGAAGCGGAGTCTGGA3′	60°C
**Telomere length measurement**				
telg<br>telc	-	79	5′ACACTAAGGTTTGGGTTTGGGTTTGGGTTTG GGTTAGTGT3′<br>5′TGTTAGGTATCCCTATCCCTATCCCTATCCCTA TCCCTAACA3′	59°C
ALB				
albugcr2<br>albdgcr2	-	98	5′CGGCGGCGGGCGGCGCGGGCT GGGCGGCC ATGCTTTTCAGCTCT GCAAGTC3′<br>5′GCCCGGCCCGCCGCGCCCGTCCCGCCGAGC ATTAAGCTCTTTGG CAACGTAGGTTTC3′	85°C
